# CFSP: a collaborative frequent sequence pattern discovery algorithm for nucleic acid sequence classification

**DOI:** 10.7717/peerj.8965

**Published:** 2020-04-20

**Authors:** He Peng

**Affiliations:** School of Information Science and Engineering, Xiamen University, Xiamen, Fujian, China

**Keywords:** Mutational information mining, Long range correlation, Sequence feature extraction

## Abstract

**Background:**

Conserved nucleic acid sequences play an essential role in transcriptional regulation. The motifs/templates derived from nucleic acid sequence datasets are usually used as biomarkers to predict biochemical properties such as protein binding sites or to identify specific non-coding RNAs. In many cases, template-based nucleic acid sequence classification performs better than some feature extraction methods, such as N-gram and k-spaced pairs classification. The availability of large-scale experimental data provides an unprecedented opportunity to improve motif extraction methods. The process for pattern extraction from large-scale data is crucial for the creation of predictive models.

**Methods:**

In this article, a Teiresias-like feature extraction algorithm to discover frequent sub-sequences (CFSP) is proposed. Although gaps are allowed in some motif discovery algorithms, the distance and number of gaps are limited. The proposed algorithm can find frequent sequence pairs with a larger gap. The combinations of frequent sub-sequences in given protracted sequences capture the long-distance correlation, which implies a specific molecular biological property. Hence, the proposed algorithm intends to discover the combinations. A set of frequent sub-sequences derived from nucleic acid sequences with order is used as a base frequent sub-sequence array. The mutation information is attached to each sub-sequence array to implement fuzzy matching. Thus, a mutate records a single nucleotide variant or nucleotides insertion/deletion (indel) to encode a slight difference between frequent sequences and a matched subsequence of a sequence under investigation.

**Conclusions:**

The proposed algorithm has been validated with several nucleic acid sequence prediction case studies. These data demonstrate better results than the recently available feature descriptors based methods based on experimental data sets such as miRNA, piRNA, and Sigma 54 promoters. CFSP is implemented in C++ and shell script; the source code and related data are available at https://github.com/HePeng2016/CFSP.

## Introduction

Feature extraction from nucleic acid sequences is an essential pre-requisite for nucleic acid sequence classification. Feature extraction is the process of representing raw sequence data in vector form that machine learning algorithms recognize. Ideally, feature vectors capture the essential characteristics of the original sequence while also being compacted. Formal compaction is convenient for predicting model building. Feature extraction methods, or tools for sequence analysis, such as miRNAfe  (Yones et al., 2015), which were developed to extract features from RNA sequences, have been studied in the past. Zhou & Liu (2008) proposed a feature extraction method for predicting protein-DNA interactions. Prediction models perform well when there is enough information to describe the original sequence  (Littlestone, 1988). The development of next-generation sequencing and the availability of large experimental data sets provide sufficient descriptive information about original sequences. However, increasing data may also contain noise or irrelevant information. To better extract a pattern from the data set, a new approach based on frequent sub-sequences was developed, which are gap-less fragments that frequently appear in nucleic acid sequences. In sequence data with good quality, noise consists of a small segment of the original sequences; thus, in a data set with a low error rate, the frequent sub-sequences contain less incorrect information. Furthermore, extracting a set of frequent sub-sequences from a given sequence is an efficient way to extract features for the sequence prediction model. Compared with N-gram sequences (Tomović, Janičić & Kešelj, 2006), these frequently extracted sub-sequences, which are used as input features for the prediction model, have greater flexibility of length. The number of N-gram sequences increases exponentially as the length of the sequences increases (Lesh, Zaki & Ogihara, 1999). Infrequent N-gram fragment sequences, which may contribute little to improve prediction accuracy (Cheng et al., 2007) may also result in over-fitting. In the k-spaced (element pair) feature extraction method, a pair of single bases with a fixed space  (Li, 2010) is used to perform features matching. This method performs well in applications such as protein methylation modifications. However, one shortcoming is that the single base is inadequate to capture enough sequence traits. In SVM-Prot  (Cai et al., 2003), a web-based tool to perform functional classification for sequences, integrating known protein family information is necessary. Thus, SVM-Prot is suitable for protein sequences. Position weight matrices also can be used as features, e.g., when a DNA motif is obtained by a motif detecting tool, such as meme, PWMEnrich R software  (Stojnic & Diez, 2014) is used to enrich the motif in the training data set. The shortcomings of these methods is that the length of the DNA motif is fixed, and long-range correlation patterns, such as frequent co-occurrence motifs with large distance, cannot be captured.

A type of frequent sequence based descriptor was introduced, which is obtained by generating a set of all frequent sub-sequences for a data-set. Each sub-sequence includes mutation profiles. A trie  (De La Briandais, 1959; Liu et al., 2003) is employed to enumerate all frequent sequences with a frequency higher than a certain threshold. Frequent part-sequences are repeatedly filtered to ensure that none of them are present in the set as parts of a longer sequence. Such sub-sequences are called Closed Frequent Sub-sequences. The frequent partial sequences are combined to increase the expressiveness of the feature space. Compared with single frequent sub-sequences, frequent sub-sequence tuples capture more underlying semantics from the original sequences. The arrangement of frequent partial sequences results from the combinations of the frequent sub-sequences. Compared with the original purpose for closed frequent pattern mining  (Prabha, Shanmugapriya & Duraiswamy, 2013), a revised form is used to generate combinations of frequent sub-sequences in which duplicate sub-sequences are allowed in each combination. By using this type of closed ”frequent pattern mining ” method, tuples with frequent partial sequences are obtained, thereby enabling gaps among frequent partial sequences.

In most cases, if a slight difference between the sub-sequences exists in the same positive data set (sequences with similar bioactivity), this subtle difference pattern should be preserved too. Thus, strict sub-sequence matching is not recommended for biological sequence descriptors. Mutational information, which also stores the frequency of the mutation type (insert, delete, or substitute) in each sub-sequence is attached. Every single frequent sub-sequence, which is sensitive enough to capture small variances, is attached to a mutation profile. At this time, tuples with frequent partial sequences have sufficient information that can be used as a descriptor for classification models, such as microRNA identification, DNA binding site prediction, and piRNA identification. Experimental results show that significant improvement in classification accuracy is achieved by using frequent partial sequences as features.

## Method

### Frequent sub-sequence tuples generation

In this section, an explanation is presented about how to generate frequent sub-sequence tuples from nucleic acid sequences and then use the numerical values of tuples as an input vector for sequence analysis. The steps used to generate frequent sub-sequence tuples are as follows:

1. All frequent subsequences (parts of sequence), which repeatedly occur in sequences of the dataset, are found. The adjustment of a suitable frequent threshold depends on the heterogeneity of the dataset.

2. A combination of single frequent sequences is obtained, and the frequent sequence arrangement is derived from the combination by re-scanning original sequences.

3. The mutation profile of each frequent sub-sequence is obtained in the frequent sub-sequence tuple. The mutation profile is a kind of format to record mutational information.

4. Frequent sub-sequence tuples are used as features to generate descriptors for sequence classification.

**Figure 1:**
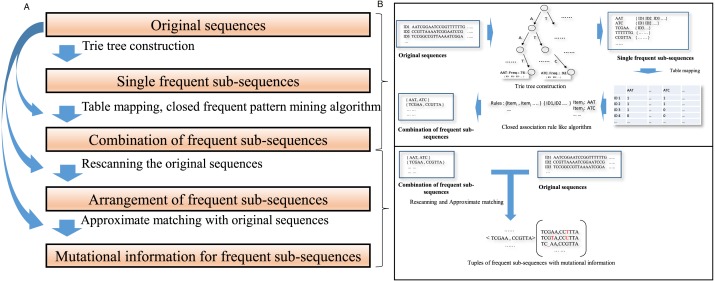
A schematic diagram for the algorithm description. (A) A concise algorithm flow for frequent collaborative sequences pattern detection is illustrated. (B) The crucial processing part of the algorithm is shown in a straightforward approach.

The schematic describing the algorithm is shown in Fig. 1. The details of those steps are as follows:

(1) Mine all frequent sub-sequences from a data set of sequences.

A trie tree data structure (De La Briandais, 1959; Yao, 1975) is constructed to identify individual frequent sub-sequences in the whole dataset. Trie, a kind of prefix tree, is a common string indicated by a node, where each node is associated with the list of IDs of the sequences in which the common string appears. A sub-sequence is acquired by in-depth traverses within the trie prefix tree. For two substrings, if one is part of another and they occur in the same sequences, only the super-string is retained; the substring is eliminated. The frequency of sub-sequence appearances in the original sequences should exceed a threshold that depends on the heterogeneity of the original data. The number of frequent sub-sequences depends on this threshold.

**Figure 2:**
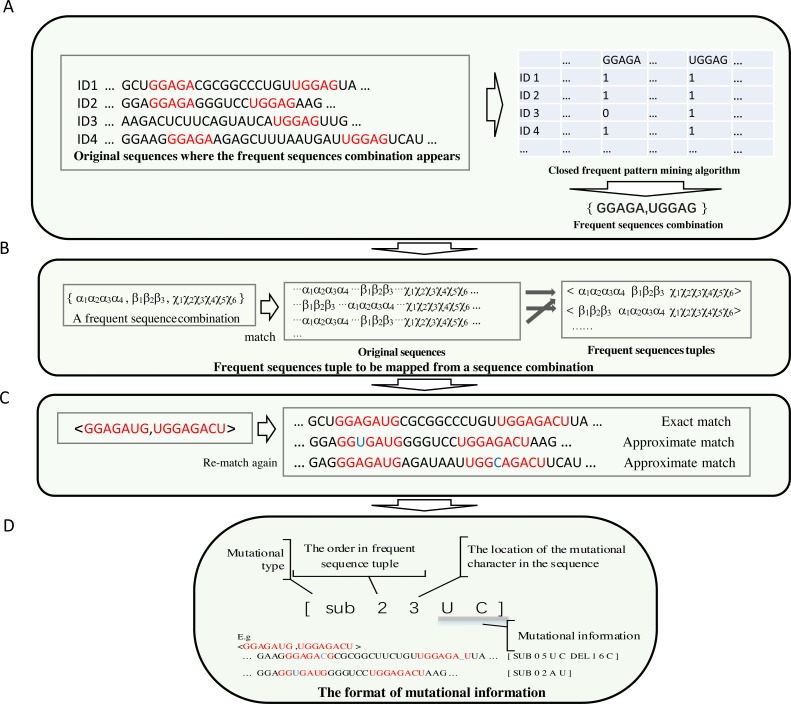
The demonstration of the algorithm in detail. (A) Each frequent sequence maps to a column of a table. Each sub-sequence is represented by a symbol, and the frequent sequences combinations were derived. (B) The order of each sub-sequences in the combination of each frequent sequence is obtained by re-scanning the original sequences. (C) The achievement of the frequent sequences approximate matching (implemented by non-deterministic finite automaton algorithm) is shown. (D) Compact data structure for recording mutation information. The first digit represents the type of mutation, which can be either sub(substitution), ins (insertion) or del (deletion). The number for location begins from zero.

To make the most frequent sequences appear in a specific position in a sequence, long, frequent sequences above a threshold are retained. Each frequent sub-sequence is treated as a symbol. A frequent sub-sequence that appears in a sequence is represented as a column in a table. The process is demonstrated in Fig. 2A.

(2) The combinations of correlated frequent sub-sequences are obtained from a table derived from the search for frequent sub-sequences in the original sequences. The order of the frequent sub-sequences in a combination result from the re-scan of the original sequences.

In most cases, thousands of frequent sub-sequences are identified. Most of the elements in the resulting table are zero; few are non zeros. Through frequent sub- sequences matching and scanning from original sequences, a table is created. Frequent sub-sequence combinations are generated from this table via a kind of revised closed frequent pattern mining algorithm (See Fig. 2A).

Through the identification process (Fig. 2B), subsequently, the frequent sequence combinations are matched via scanning the original sequences. By scanning, information about the order of frequent sequences is recorded in mapping, or, after that, if the information about this frequent sequence permutation has already been reported, the frequency of this permutation increases by one.

(3) In this step, the mutation profile of each sub-sequence in each frequent sub- sequence arrangement is obtained.

A length filter is integrated into the frequent sequence selection process. Long frequent sequences accurately represent biological functions. However, because in the positive data set (the sequences that resemble functional biological properties), if a small change in a long sequence occurs frequently, this mutational type may have little effect on biological functions. The prediction model should not ignore the small changes completely. Therefore, exact matching of frequent sequences is unsuitable as a descriptor for predicting biological functions. This shortcoming can be overcome by attaching a mutation profile to each sub-sequence in each permutation. This kind of mutation profile is determined via an approximate string matching with the original sequences. In approximate matching, limited insertions, deletions, and substitutions are permitted, as shown in Fig. 2C. In this approximate string matching, the Edit-distance Metric Method is used.

Approximate matching is implemented by a non-deterministic finite automaton (GNU C Library, 1983; Rabin & Scott, 1959). In this way, a group of states represents a matching string. Different automaton translations between states indicate distinctive events, such as exact matching, matching by substitution, deletion or insertion. Each event has a score; the score of matching events is zero; the scores of substitution, deletion and insertion events are less than zero. The strategy (branch) that traverses the approximate match with the maximum score is retained. If the maximum score is below a threshold, it is ignored as a matching failure. This strategy indicates that only limited deletion and insertion mismatch events are allowed.

The original data set is re-scanned for each frequent sub-sequence permutation. An extended data structure is used to store the mutational information. The details of recording the mutational information (mutational type and where the mutation occurs, etc.) are illustrated in Fig. 2D. The frequency of each mutation type is recorded before each entry of mutational information. The frequency of tuple of frequent sequences is how often this tuple of frequent sequences that match exactly has occurred in the entire data set that is recorded at the beginning of all entries of mutational information for this tuple.

(4) The frequent sub-sequence tuples are used as features to generate a descriptor vector for each sequence for sequence classification.

There are many popular methods to extract features, e.g., k-mer (Dubinkina et al. 2016) and CKSAAP (Zhao et al. 2012). The proposed method, which extracts features using frequent sequences tuples, captures more information about the sequence than other methods. To map a sequence to a descriptor vector, sub-sequences, along with their mutation profiles, are used. If a sub-sequence exactly matches a sequence, then the value of the descriptor position for these frequent sequences tuple is 1.0. If a frequent sequence tuple roughly matches a sequence, then its mutation record is used to calculate the value of the descriptor at this position that corresponds to this type of mutation. The value of the feature descriptor is the ratio of the frequency of occurrence of this mutation type to the frequency of the frequent sequences tuple.

For example, consider a frequent sequences tuple <GGAGAUG, UGGAGACU >, the frequency of occurrence of this tuple of frequent sequences in the original dataset is 16. For mutational information <GGAGACG, UGGAGA_U >, [SUB 0 5 U C DEL 1 6 C], the occurrence frequency of this mutation type for this tuple of frequent sequences is 7.

Exact match:

For sequence ... GGAGGAGAUGGGGUCCUGGAGACUAAG ... if the frequent sequences tuple is an exact match, the feature value is 1.0.

Approximate match:

For a sequence ... GGAGGAGACGGGUCCUGGAGA_UAAG ... if the tuple of frequent sequences approximately matches, and this mutational information can be found, then the feature value is 7/16, described in Eqs. (1) and (2) below.

The sequence *S*_*i*_ is encoded by

(1) \begin{eqnarray*}c({S}_{i},FS{S}_{j})= \left\{ \begin{array}{@{}ll@{}} \displaystyle 1 &\displaystyle if\,FS{S}_{j}\,\,exactly\,matches\,{S}_{i}\\ \displaystyle \frac{frequency\,of\,M{T}_{jk}}{frequency\,of\,FS{S}_{j}} &\displaystyle if\,FF{S}_{j}\,\,approximatelymatches {S}_{i},and\,the\,mutation can befound\,in M{T}_{j}\\ \displaystyle 0 &\displaystyle Otherwise \end{array} \right. .\end{eqnarray*}

(2) \begin{eqnarray*}\phi ({S}_{i})= \left[ \begin{array}{@{}c@{}} \displaystyle c({S}_{i},FS{S}_{1})\\ \displaystyle c({S}_{i},FS{S}_{2})\\ \displaystyle \cdots \\ \displaystyle c({S}_{i},FS{S}_{n}) \end{array} \right] .\end{eqnarray*}

*S*_*i*_: The predicted sequence.

*FSS*_*j*_: The frequent sequences tuple j.

*MT*_*j*_: The mutation types for the frequent sequences tuple j.

*MT*_*jk*_: A matched mutation type k for the frequent sequences tuple j.

*ϕ*(*S*): A mapping from the original predicted sequence *S* to a vector.

### The details of frequent sequences combination generation implementation

Combinations of frequent sequences, such as the closed pattern mining algorithm, are generated (Prabha, Shanmugapriya & Duraiswamy, 2013). Each frequent sequence is treated as an individual symbol, and their combinations are considered as a composite symbol, which frequently appears in the original dataset. This frequent composite symbol combination must be closed (If these combinations appear in the same nucleic acid sequences, only the maximal one is kept).

**GGAGAUG**: *α*

**UGGAGACU**: *β*

*ID*_*i*_: ... GGA**GGAGAUG**GGGCC**UGGAGACU**AAG ...

*ID*_*j*_: ... GGA**GGAGAUG**GGGCC**UGGAGACU**AAG ...

If the symbol combinations {*α*}, {*β*}, {*α*, *β*} appear in the same sequences {*ID*_*i*_, *ID*_*j*_}, only the composite symbol {*α*, *β*} with maximum length is retained.

Unlike the traditional closed frequent pattern mining algorithm, duplicate symbols are permitted in symbol combinations. For a symbol combination having duplicated symbols, additional symbols are introduced to translate the original symbol combination into a symbol combination with unique symbols.

For a symbol repeated n times {*α α α*...} in a symbol combination, the duplicate symbols are translated into {*α*,2 *α*,…,n*α*}, so that symbol duplicates of different lengths are treated differently.

That is for example

**GGAGAUG**:

**UGGAGACU**:

GGA**GGAGAUG**GU**GGAGAUG**CC**UGGAGACU**AG... {*α*, *α*, *β*}- >{*α*,2 *α*, *β*}

The frequent symbol combination enumeration is implemented by a depth-first search based on stack architecture instead of a recursive calling. In this way, This ensures deep recursion for a large number of symbols .(Details of the algorithm implementation are given in Supplementary Information A).

### How to build a predictive model with commands

If the CFSP software is installed correctly, and all commands are available, constructing a prediction model from the data set is straight-forward. The command to obtain the data set, which is necessary to generate the features is described as follows:

FeatureGen FeatureDataSet FeatureFile

FeatureDataSet is the name for fasta formatted file for feature generation. The command produces two files: FeatureFile and FeatureProfile. A user needs two labeled datasets: positive labeled and negatively labeled.

The following command generates a labeled sparse matrix format file (*.libsvm).

libsvmGenWithFeature FeatureFile.Feature FeatureFile.FeatureProfile PositiveDataset Negativedataset OutFileName

This sparse matrix format file can be loaded into the statistical analysis system R by using the ‘e1071’ package. The construction of a prediction model can then be easily accomplished with the R-script

motifTools ToPSSM FeatureFile FeatureProfileFile OutPutPSSMFile

This command is used to transform the mutational information format to PSSM (Position Specific Scoring Matrix) format. FeatureFile is a file storing frequent sequences tuples; FeatureProfileFile is a mutational information file, and the OutPutPSSMFile is a PSSM format output file.

## Results and Materials

This method is applied to the following three different biological sequence prediction problems: Namely, (i) miRNA identification, (ii)sigma-54promoters identification, (iii) piRNA identification. Each sequence, with feature mapping, is mapped to a vector consisting of Boolean or numerical values. Used for classification are libsvm and liblinear software packages (Chang & Lin, 2011; Fan et al., 2008), which are encapsulated in R language scripts. Used for the comparison feature extraction method is BioSeqClass of R software package (Li, 2010). For easy reproduction of results, the data set and used parameters are given in Supplementary Information B). Besides, this algorithm is applied to find a protein binding pattern from the public ChIP-seq data set. The results are provided in the figures for this experiment. Because the binding sites of a biological protein in a whole genome are always heterogeneous, the frequent threshold for filtering is very low. The patterns with a frequency ratio greater than 0.00125 are preserved. These patterns contain collaborative repeat sequences or conserved sequences. A mutation profile, recording the mutational information of each base, which resembles the position weight matrix, is added to the frequent sequence. In the final experiment, this method is applied to the field of protein sequence classification. (The data set and detailed parameter settings can also be found in Supplementary Information B).

### miRNA identification

The objective of this classification task is to distinguish real miRNA precursors from pseudo ones. The tuples of frequent sequences are selected as features. The model of libsvm (Chang & Lin, 2011) is chosen as the prediction model. The proposed method, which extracts features using frequent sequences tuples, captures more information about the sequence than other methods. Frequent sequence tuples were derived from a hairpin sequence data set containing 46,231 sequences downloaded from miRBase (Kozomara & Griffiths-Jones, 2013) A total of 2,719 frequent sequences tuples were obtained via CFSP algorithm. Species-specific training data sets come from (Peace et al., 2015), and 10-fold cross-validation is employed. The number of sequences for each species data set is shown in Table 1. The results are listed in Table 2. In Table 3, the results of 10 -fold ROC (One fold for test data set. Nine folds for training data set) are illustrated. K-mer and gkmSVM (Ghandi et al., 2016) are selected as comparison methods. To deal with an unbalanced data set, a weight is assigned that corresponds to the positive or negative label to each sequence in the training data set. The weight in the positive data set is 1 and in the negative data set, the weight is the inverse ratio between the size of the negative data set and the size of the positive data set. Then the SVM method is used as a predictive model. Therefore, this method is designated the weighted SVM. To justify which prediction model is suitable for the prediction of microRNA, the combinations of CFSP and various methods of machine learning (Weighted SVM, Random Forest, Neural Network) are tested. Three unbalanced datasets of species are selected. The Random Forest method uses the R-package randomForest , and the Deep Learning R-package Keras is selected as the tool for the neural network’s approach. The results are listed in the Table 4.

**Table 1:** The data size for each species.

Species	Positive data size	Negative data size
*Anolis carolin ensis*	282	500
*Arabidopsis thaliana*	298	457
*Drosop hila pseudo obscura*	691	2,094
*Arabid opsis lyrata*	691	1,437
*Drosop hila melano gaster*	238	443
*Epstein barr virus*	691	2,310
*Xenopus tropicalis*	691	2,023

**Table 2:** The results of average accuracy of 10-fold of miRNA identifying for various species.

Species	CFSP	k-mer	gkmSVM
*Anolis carolin ensis*	81.46%	64.07%	65.95%
*Arabidopsis thaliana*	90.20%	85.70%	90.79%
*Drosop hila pseudo obscura*	93.18%	91.20%	77.43%
*Arabid opsis lyrata*	84.87%	77.73%	70.28%
*Drosop hila melano gaster*	93.39%	86.34%	81.10%
*Epstein barr virus*	93.04%	89.80%	89.02%
*Xenopus tropicalis*	85.08%	75.86%	55.67%

**Table 3:** The results of ROC of miRNA identifying for various species.

Species	CFSP	k-mer	gkmSVM
*Anolis carolin ensis*	0.90	0.73	0.78
*Arabid opsis thaliana*	0.86	0.68	0.93
*Drosop hila pseudo obscura*	0.97	0.76	0.89
*Arabid opsis lyrata*	0.87	0.75	0.80
*Drosop hila melano gaster*	0.97	0.81	0.87
*Epstein barr virus*	0.92	0.72	0.95
*Xenopus tropicalis*	0.83	0.78	0.74

**Table 4:** The results of CFSP combining with various machine learning methods.

Species	Method	Accuracy	ROC
*Drosophila pseudoobscura*	Weighted svm	93.52%	0.98
	Random Forest	94.24%	0.97
	Neural Network	92.81%	0.97
*Epstein barr virus*	Weighted svm	92.00%	0.97
	Random Forest	91.00%	0.99
	Neural Network	89.67%	0.95
*Xenopus tropicalis*	Weighted svm	79.33%	0.86
	Random Forest	80.44%	0.83
	Neural Network	78.97%	0.86

**Table 5:** The results of average accuracy (10-fold cross validated) for Sigma-54 promoter prediction.

Method	Average accuracy	Average ROC
CFSP+svm	79.82%	0.89
k-mer+svm	75.16%	0.84
CFSP+Random Forest	82.96%	0.93
k-mer+Random Forest	79.33%	0.86

### Identification of sigma-54 promoters

**Figure 3:**
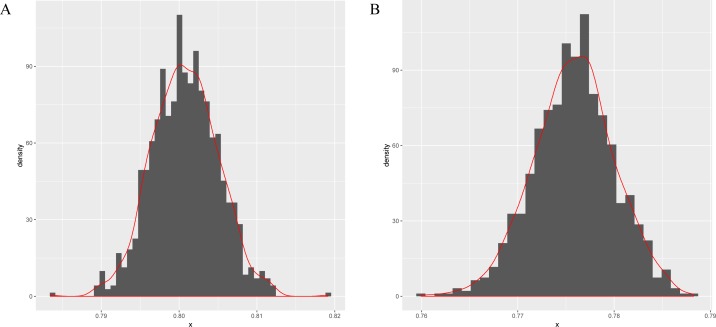
The bootstrap statistics graph for CFSP method (A) and the k-mer way (B).

Next, this model is applied to sigma-54 promoter prediction. In the data set obtained from (Lin et al., 2014), there are 161 positive sequences and 161 negative sequences. The results of the average accuracy of the prediction results for the frequent sequences tuple+(SVM, random forests (Wright & Ziegler, 2015) and compared combination methods k-mer+(SVM, random forests) are listed in Table 5. As seen in Figs. 3A–3B, the data set is randomly split into test and training data sets in a ratio of 1:9 for 256 times to obtain the predicted results. The bootstrap statistics (Harman & Kulkarni, 1998) graphs are obtained via sampling 1,000 times with replacements from the original 256 predicted results (for CFSP method and the k-mer method).

**Table 6:** The results of piRNA prediction.

Method	Sp(%)	Sn(%)	Acc (%)
k-mer	98.4	52.04	75.22
Pibomd	89.76	91.48	90.62
Asysm-Pibomd	96.2	72.68	84.44
CFSP	89.11	89.17	89.12

### piRNA identification

There were 51,664,769 mouse piRNA sequences collected from the piRBase (Zhang et al., 2014). Due to limited computational resources, five million piRNA sequences were randomly selected for tuples of frequent sequences extraction. Liblinear (Fan et al., 2008) was used to design machine-learning models. The training data set and the test datasets were collected from (Zhang et al., 2014) reference. In the training data set there are 5,000 known piRNA sequences and 5,000 non-piRNA sequences. The frequent sequences tuples from 5,000 known piRNA sequences combined with 1,419 frequent sequences tuples are treated as feature descriptors. In the test data set, there are 2,500 known piRNA sequences and 2,500 non-piRNA sequences. The piRNA identifying methods (Pibomd, Asysm-Pibomd) from (Liu, Ding & Gong, 2014), which have shown excellent results, are chosen as comparison methods. The prediction results of frequent sequences tuples and comparison methods( k-mer, Pibomd, Asysm-Pibomd) are listed in Table 6.

**Figure 4:**
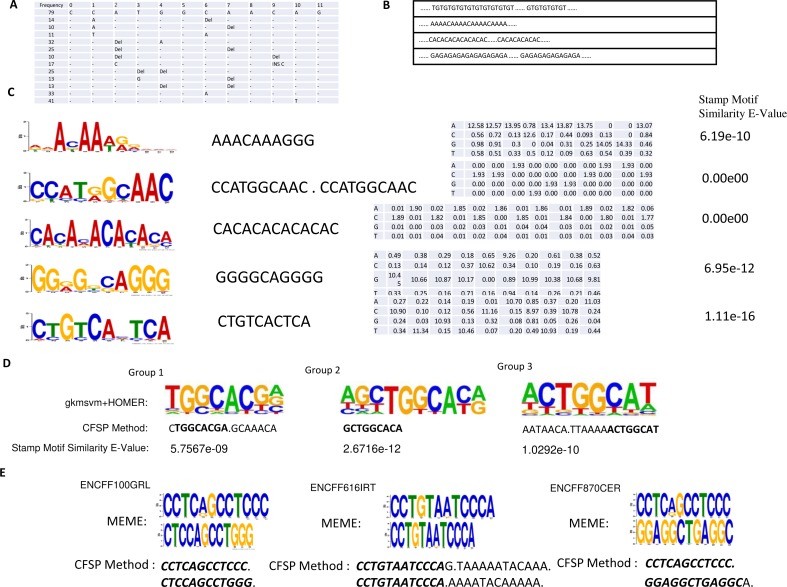
(A) One conserved sequence, which occurs 79 times in 46,264 binding site peaks from the ChIP-seq data-set. The mutation profile of this conserved sequence is illustrated, where ’_ ’ indicates this base is unchanged; DEL indicates this base is lost; INS *X* indicates a new base *X* is inserted in front of this base. (B) Several repeated elements patterns are listed. (C) In the first column, the top five DNA motifs, mined by meme-chip tools (Machanick & Bailey, 2011) are illustrated. The resemblant conserved sequences, found by the CFSP algorithm are listed in the second column. In the third column, the position-specific scoring matrices, which are transformed from mutational information are listed. The similarity between meme motif and resemblant conserved sequence with PSSM format was calculated via a stamp motif comparison tool (Mahony & Benos, 2007). The *E*-values for the similarity of those pairs is displayed in the fourth column. (D) One motif is selected in each group clustered by gkmsvm descriptors, and the corresponding motif found by the CFSP algorithm is listed below. (E) There are additional datasets (File No: ENCFF100GRL, ENCFF616IRT, ENCFF870CER, Target: SREBF1) collected from https://www.encodeproject.org. The top two motifs are selected in each file using meme tools, and the corresponding motifs found by our algorithm are listed below.

### Protein binding site detection from ChIP-seq data

The ChIP-seq dataset for SMARCA4 protein was collected from the NCBI GEO (Accession: GSE125033). Peak calling reveals 46,264 binding sites are collected. The minimum frequency for each frequent sub-sequence is 0.00125. The length of frequent sub-sequence is variable, but restricted to a range that must be greater than 10 bp. (There are 6,751 patterns detected preserved in Supplementary Information B). Some patterns are listed in Figs. 4A–4C. Also, a meme-chip tool was applied to find five motifs from 46,264 binding sites. The motifs, which are most similar to those five motifs, are selected from the patterns detected by CFSP method. A total of eight hours and ten minutes are consumed for five motifs finding via meme-chip tool (6 core (30 MHz), Memory 120G). In CFSP method, the extracted frequent sub-sequences consume 29 min. Then the frequent sub-sequences which are most similar to five meme motifs are selected. Mutation information and conversion to PSSM format can be accomplished within half a minute. Thus, it is evident that the CFSP method is significantly more efficient than the meme-chip tool. In Fig. 4E, three additional ChIP-seq data-sets are tested; two motifs are selected in each data-set. The corresponding motifs found by the CFSP algorithm are shown below. In addition, the more accurate method (combination of gkmsvm (Ghandi et al., 2016) and HOMER (Heinz et al., 2010)) is also used as a comparison. The sigma-54 promoters binding site data-set in ‘Method’ is selected. In the gkmSVM R package, ’gkmsvm kernel’ interface calculates the similar matrix for positive sequence data-set. The distance between two sequences is calculated from 1 - the similarity of two sequences. Using these distances, a hierarchical cluster analysis is carried out to group the sequences. Finally, three groups are selected. The sequences in each group have close proximity. With the HOMER Motif tools, the top one motif is selected in each group. The corresponding motifs, found by the CFSP algorithm, are listed below (Fig. 4D).

### Extend to protein sequences identification

The CFSP method is designed especially for classification tasks of nucleic acid sequences. In a final experiment, the suitability of the CFSP method to classify protein sequence is investigated. ProtVecX is one of the state-of-the-art methods for protein sequence embedding. In this method, the variable-length motifs are extracted via an efficient alignment-free way; then the data are used as the features of sequences (see Table 7).

**Table 7:** The data for protein sequences identification.

Data name	Training or test	Positive or negative	Data size
Biofilm	Training Data Set	Positive Sequences	1,305
	Training Data Set	Negative Sequences	1,463
	Test Data Set	Positive Sequences	145
	Test Data Set	Negative Sequences	163
Integrins	Training Data Set	Positive Sequences	100
	Training Data Set	Negative Sequences	518
	Test Data Set	Positive Sequences	12
	Test Data Set	Negative Sequences	58

The prediction results of the CFSP method compared with ProtVecX are listed in Table 8.

**Table 8:** The results of protein sequences classification.

DataSet	Method	Precison	Recall	F1
Integrins	ProtVecx(Best representation)	1	0.83	0.91
Integrins	frequent sequences tuples	1	0.91	0.97
Biofilm formation	ProtVecx(Best representation)	0.82	0.56	0.72
Biofilm formation	frequent sequences tuples	0.97	0.78	0.87

## Conclusions and Discussion

A new motif features extraction approach for the construction of prediction models was proposed. The individual frequent sequences were collected in the first step; then, frequent sequence combinations were generated by a modified version of a closed frequent pattern mine algorithm. Finally, tuples of frequent sequences were obtained via re-scanning the original sequences. The set of extracted features is used as input for miRNA identification and Sigma-54 promoter prediction. The results of the CFSP method show significant improvement. The CFSP method is superior to the k-mer methods. Although a tool specifically designed for the detection of protein binding sites, gkmSVM is also excellent for the detection of miRNAs. If mutational information is considered, it performs better. The barrier that prevents k-mer from obtaining better results is the length of the k-mer. If the segment size for k-mer is short, it causes a loss in the long-range correlation relationship. The k-spaced pair method lacks specifics for the local sequence. The advantage in the CFSP method is that a frequent sequence is a variable-length nucleotide that frequently occurs among a set of sequences. The tuples of frequent combining sub-sequences detect the long-distance correlation relationship. Judging from the results of combining the methods of the CFSP method and various machine learning methods (Weighted SVM, Random Forest, Neural Network), the random forest method appears to be slightly better; however, the evidence is not reliable due to fluctuation. The data set is not large enough, and this may be the reason that the Deep Neural Networks method is not as good as expected. Features extracted from miRBase in an unsupervised manner are used to construct various prediction models using the various train set for diverse species. This strategy is similar to one in the method (Hassani & Green, 2019) in which an unlabeled data set is used. The approach in this reference is especially useful when there is little marked training data. e.g., for newly sequenced species. In the next step, by integrating the proposed feature extraction method, this method is used to improve the predictive ability of new species miRNAs.

Performance evaluation on piRNA identification shows that frequent sequences tuple (CFSP) based prediction models perform better than Asysm-Pibomd (Liu, Ding & Gong, 2014), which is a state-of-the-art software tool based on motif feature descriptors. This result is close to the Pibomod method based on the TIRESIAS motif. One advantage of the CFSP approach over the TIRESIAS motif is that long-range correlation can be considered. If the long-distance relationship is fundamental for the classification in the sequence classification, the CFSP method performs better.

Furthermore, variable gaps exist between single frequent sequences. However, as currently implemented, if one site in a single frequent sequence is infrequent, then this single frequent sequence is divided into two pieces. If a few uncommon gaps within a single frequent sequence are allowed, more expensive computational complexity costs are induced. To make sure that infrequent sites can exist in a base unit sequence, the TEIRESIAS (Rigoutsos & Floratos, 1998) motif is integrated with a single frequent sequence as the base unit of frequent sequences tuple.

Good results of the CFSP approach in the application of nucleic acid sequence show that the CFSP method is a powerful tool for nucleic acid sequence feature extraction. Currently, the prediction model focuses only on molecular level property prediction. For the more elaborate phenotype prediction, which is related to multi-omics, a more complicated workflow and labeled data-set are necessary. DiTaxa (Asgari et al., 2018) is an approach that has competitive performance in the phenotype prediction. This method provides an excellent computational workflow for phenotype prediction and biomarker detection, taxonomic analysis. The sequence representation method (Nucleotide-pair Encoding (NPE)) algorithm in this method is similar to that in the CFSP method. The difference is that in the CFSP method, the distance between sub-sequences in each combination is unlimited. But, in most cases for miRNA, the distance between sub-sequences is short. Therefore, this advantage does not assume the central role in short RNA sequence classification.

In the CFSP method, the motif is efficiently extracted and conveniently used as a sequence feature. A tool to convert this motif format to PSSM (Position-Specific Scoring Matrix) format has been developed. But for motif visualization and interpretation, there are still some areas for improvement. In the SeqGL (Setty & Leslie, 2015) method, the group lasso regularization and k-mer feature representation are employed to identify sequences groups that discriminate between peak sequences and flanks. Subsequently, the HOMER method is applied to find motifs. One improvement of the SeqGL method is that the feature representation method (K-mer) in the SeqGL can be replaced by the CFSP method to find interpretable and visualizable motifs.

Other improvements of the CFSP algorithm are that this method mainly focuses on nucleic acid sequence motif extraction. Protein is the main component of cells and is essential to life. Understanding the protein sequence is crucial for biological processes discovery. HH-MOTiF (Prytuliak et al., 2017) is one method for short linear motifs detection. In this method, the motif root is chosen as a template to align the motif leaves using HMMs. In the CFSP method, a similar approach for implementing a fuzzy match is a non-deterministic finite automaton, which is simple but very efficient. In further work, for the protein sequence match, the usage of a more complicated non-deterministic finite automaton is necessary.

The assignment of protein sequence to function or structure is intricate; for example, protein sequences with weak sequence similarity may have very similar structures. Shallow machine learning techniques such as SVM, etc. may be insufficient. The deep learning method for natural language processing can be considered. For example, modern natural language processing technology was used in the protVec (Asgari & Mofrad, 2015) method. The CFSP method was compared with ProtVecX (Asgari, McHardy & Mofrad, 2019), which is a byte-pair encoding based protein sequence embedding method extended from protVec. Statistically, the results for this method are close to ProtVecX.

**Project home page:** https://github.com/HePeng2016/CFSP

**Operating system(s)**: linux, unix or Cygwin in windows

**Programming language**: C++, shell script

**Other requirements**: C++11 or higher

**License**: MIT License

##  Supplemental Information

10.7717/peerj.8965/supp-1 Supplemental Information 1 How to implement the frequent sequences combination generation This algorithm is a closed frequent pattern mine algorithm in which the duplicate symbols are allowed in each frequent combination.

10.7717/peerj.8965/supp-2 Supplemental Information 2 Dataset, scripts and detailed parameter setting

## Abbreviations

MEME: Multiple EM for Motif Elicitation
k-mer: All the possible sub-sequence of length k that are contained in a sequence
CKSAAP: Compositon of k-spaced Amino Acid Pairs
CFSP: Collaborative frequent sequence pattern discovery algorithm
NFA: nondeterministic finite automaton: NFA is represented formally by a 5-tuple,that can recognize the regular languages
Frequent Subsequence: The string frequently appear as substring in the original sequences.

## Additional Information and Declarations

### Data Availability

The following information was supplied regarding data availability: The algorithm, data-set, scripts and details of parameter setting are available in the Supplementary Files. CFSP is written in C++ and shell script, the source code and related data are available at https://github.com/HePeng2016/CFSP.
