# Supplementary material for: CFSP: a collaborative frequent sequence pattern discovery algorithm for nucleic acid sequence classification
Source: PeerJ. 2020 Apr 20;8:e8965. doi: 10.7717/peerj.8965 (PMC7179567; doi:10.7717/peerj.8965)
Supplement: Supplemental Information 1 — This algorithm is a closed frequent pattern mine algorithm in which the duplicate symbols are allowed in each frequent combination. [file peerj-08-8965-s001.pdf]

Supplementary material A

In this section, we introduce a novel way to implement closed frequent pattern mining.

A frequent closed itemset in a data set is defined that there exists no superset that has the same support count as this frequent itemset. The equivalent definition is that if some frequent sets appear in certain transactions, only the maximal one is retained.

The proof of relevance definition and lemma are given before introducing how to implement the frequent closed Itemset algorithm.

The frequent sets are enumerated via prefix tree traveling. A path from the leaf node to root node encodes a frequent set. For example, set A can be represented by the sequence $< \alpha_1, \alpha_2 \ldots, \alpha_d >$.

Let $A = \{\alpha_1, \alpha_2 \ldots, \alpha_d\}$ be a label set that indicates each element in a frequent set, and elements in label set are partially ordered with linear order.

If any element is unique in set A with a linear order, there is a unique ordered list for set A within this linear order.

If there are duplicate elements in a set, we use an additional label to translate the set into a unique sequence. For an element duplicate *n* times {e, e, ..., e}, we use label sequence <e,2e...,*ne*> to indicate this set.

<e> ->{e} One element
<e,2e> -> {e, e} Two elements
<e 2e 3e> , ->{e, e, e} Three elements
...

A sequence $\mathbf{x} = < \alpha_1\, \alpha_2 \ldots, \alpha_m >$ is a sub-sequence of another sequence $\mathbf{y} = <b_1, b_2 \ldots, b_n >$, denoted by $x \subseteq y$, if and only if $\exists$ $i_1, i_2, \ldots, i_m$, such that $i_1, i_2, \ldots, i_m < n$ and $\alpha_1 = b_{i1}$, $\alpha_2 = b_{i2}, \ldots, \alpha_m = b_{im}$. We also designate **y** is a supper-sequence of **x**.

We define the recursive linear order of ID sequences for all subsets as follows:

For two ID sequences, $\alpha = <i_1, i_2 \ldots, i_N>$, $\beta = <j_1, j_2 \ldots, j_N>$ indicate two subsets.

If $j_1 > i_1$ then $\beta \succ \alpha$.
If $j_1 < i_1$ then $\beta \prec \alpha$.
If $j_1 = i_1$, then the ID subsequence $< i_1 >$ of α equals the ID subsequence $< j_1 >$ of β.

If ID subsequence $< i_1, i_2, \ldots, i_n >$ of α equals ID subsequence $< j_1, j_2, \ldots, j_n >$ of β, then if $j_{n+1} > i_{n+1}$ and then $\beta \succ \alpha$, if $j_{n+1} < i_{n+1}$ and then $\beta \prec \alpha$,if $j_{n+1} = i_{n+1}$ then ID subsequence $< i_1, i_2, \ldots, i_{n+1} >$ of α equals ID subsequence $< j_1, j_2, \ldots, j_{n+1} >$ of β.

A n-tuple binary numeral encodes an ID sequence. As demonstrated in **Table 1**, for a set A, if an element belongs to A, then the *i*th term of this n-tuple binary numeral is1, or if this element does not belong to A, then the *i*th term of this n-tuple binary numeral is 0.

Table 1 - Scheme of encode

1 subsets	*2 labeled sequence*	*3 Label ID sequence*	*4 n-tuple binary*	*5 Integer of n-tuple binary*
$\{ x_3 \}$	$< x_3 >$	< 3 >	100	8
$\{x_1 \ , x_3\}$	$< x_1 \quad x_3 >$	< 3, 1 >	101	9
$\{x_2 \ , x_3\}$	$< x_2 \quad x_3 >$	< 3, 2 >	110	10

The linear order of a binary numeral is a lexicographic ordering.

Proposition 1. For two subsets, ν and μ, if ν is a subset of μ , μ contains ν, then $\mu \succ \nu$.

Proof: A binary numeral for ν is n, and a binary numeral for μ is m. Because ν is a subset of μ , if a term in one position of n is 1, then the term in this position of m must also be 1. Then m is greater than n; therefore, $\mu \succ \nu$.

Then the enumeration of the subsets for a set is achieved by enumerating all subsequences of the sequence for this set.

To accomplish enumeration, we construct a prefix tree for a set A, and assign each node in this prefix tree to a label ID. We define a constraint that any node ID is smaller than its left sibling node ID and its father node ID.

Case1:

As shown in **Figure S1**. (1), if a node is the first node of a node's children in the left order, then this node ID is larger than its father node ID. If this node is the root, then this node ID is the largest one.

Case2:

As shown in **Figure S1**. (2), if a node is not the first child of node in the left order, this node ID is one larger than its left sibling.

A path composed by IDs which begins with the root node ID and ends with this node ID is an ID sequence representing a subset.

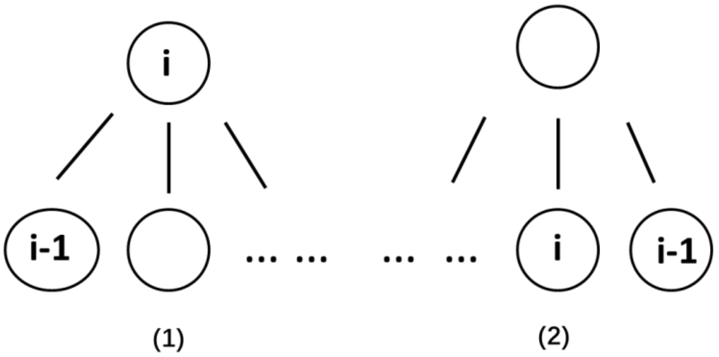

Figure S1 Two cases for search space

Proposition 2. When performing a depth-first search on a prefix tree, nodes are traveled in inverse lexicographic order.

Proof:

For two nodes, α, β, if the ID path α is part of ID path β, then β ≻α, and β must be traveled before α in the depth-first search. If ID path α is not part of ID path β, find the maximal prefix sequence for those two paths : < p_1, p_2…, p_k >, where k is the length of the prefix sequence.

The ID sequence of Path α is < p_1, p_2 …,p_k, i_{k+1}, i_{k+2} … >.

The ID sequence of Path β is < p_1, p_2…, p_k, j_{k+1}, j_{k+2} … >.Comparing the k+1 th node ID of Path α with Path β, if $i_{k+1} < j_{k+1}$ then node j_{k+1} is the left brother node of node i_{k+1} and, node j_{k+1} must be traveled before node i_{k+1}. In summary, the depth-first search order is an inverse lexicographic order.

The transaction ID list (TID-list) for an item set is the IDs of the transactions in which this item set is found.

We initially assign each item a TID-list. Then the TID-list for an item set is determined by intersecting the TID lists of items from this set.

For example, the TID list for item A is {3,4,7}, the TID list for item B is {3,4,6} and the TID list for item set {A, B} is {3,4}.

Each node in the prefix tree represents an item set. Then each node is assigned a TID list for this item set.

Proposition 3. For item sets with the same TID list, there is only one maximal item set.

Proof:

It is because if there are two maximal item sets (A and B), then the united set of A and B has the same TID list with A and B. It is a paradox that either A or B is a maximal item set within this TID list.

Proposition 4. In a depth-first traveling prefix tree, if the TID list for a node is not equal to the TID list of any children for this node, and the node with this TID list was first reached (Leaf node travel is unnecessary; this node may be on the fly), then the item set for this node is a closed frequent item set, i.e. a maximal one with this TIDlist

Proof:

The reason is that in depth-first searching on a prefix tree, nodes travel in an inverselexicographic order. Any offspring of this node will travel before this node. If there is no TID list for any offspring that is equal to this node's TID list, and there is not a node with this TID list that has traveled before, then there is no super itemset of this item set with this same TID list, because all super item sets of this item set is encoded in offspring nodes of this node. Thus, this item set is the maximal one with this TID list. An example showing the maximal item set is given in **Figure S2**.

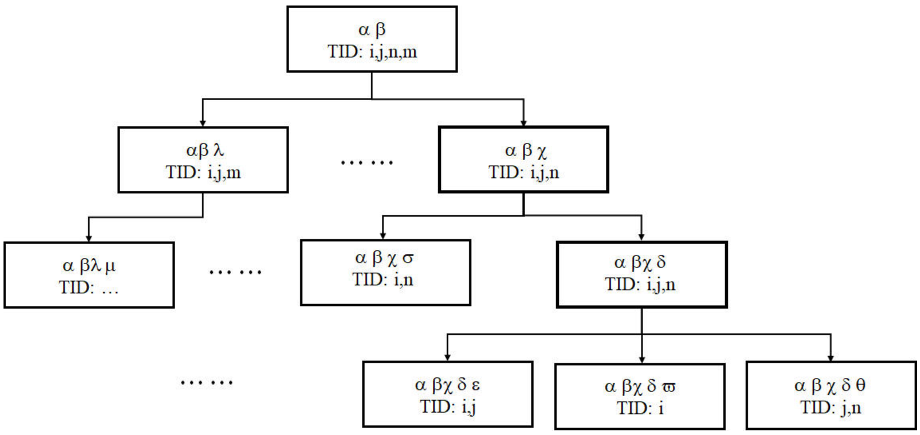

Figure S2 The strategy to find maximal one with a TID list

Proposition 5. If there are two item sets (A, B), one is a part of another (A≻B), and they have the same TID list, for any offspring node, a_i, of superset node, A, there is an offspring node, b_j, of another node, B, satisfying that b_j is a part of a_i, and they have the same TID list.

Proof:

If $< i_{b1}, i_{b2} \ldots, i_{bn}>$ is the ID sequence for node B, then any offspring of node B can be indicated by $< i_{b1}, i_{b2} \ldots, i_{bn}, p_{bn+1}, p_{bn+2} \ldots>$ and the $< p_{bn+1}, p_{bn+2} \ldots>$ is a postfixID sequence.

If $< i_{\alpha1}, i_{\alpha2} \ldots, i_{\alpha n}>$ is the ID sequence for node A, then for any offspring node b_j of node *B,* the ID sequence of b_j is $< i_{b1}, i_{b2} \ldots, i_{bn}, p_{bn+1}, p_{bn+2} \ldots>$. We add a postfix ID sequence of b_j to ID sequence of node A to construct an offspring node a_i of node A, i.e. the ID sequence of a_i is $< i_{a1}, i_{a2} \ldots, i_{an}, p_{bn+1}, p_{bn+2} \ldots >$. Because the item set for node A is a part of the item set for node B, and they have the same TID list and ID sequences, then a_i and b_j have the same postfix ID sequence so b_j is a part of a_i and they have the same TID list. An example is given in **Figure S3**.

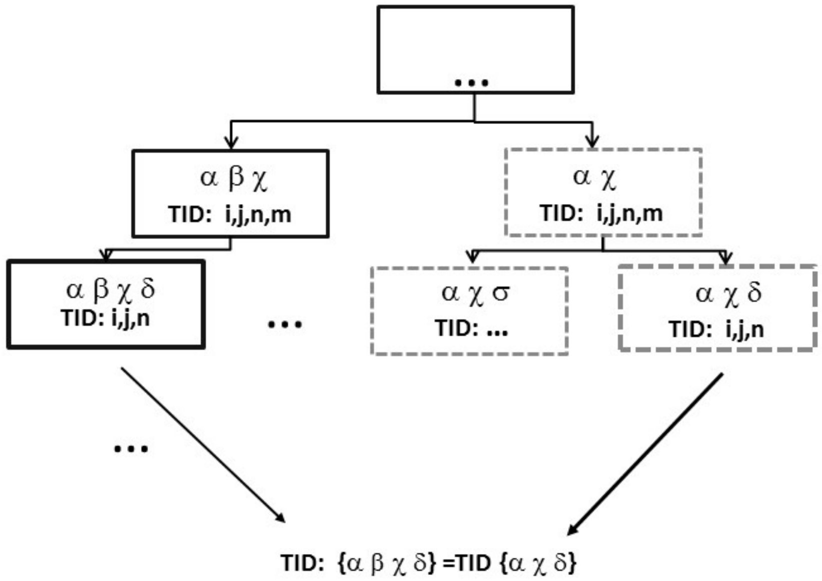

Figure S3 The nodes in gray is maximal one with TID list {i, j,n,m}.

Because TID {α β χ} = TID {α β}∩ TID {χ} and TID {α β χ δ} = TID {α β χ }∩TID {δ} , so TID { α χ }=TID{ α β χ} =>TID{ α χ δ }=TID{ α β χ δ }.

We construct a TID-list library to store the TID-lists of the nodes that have traveled.

The pseudocode is listed as follows

Algorithm 1: The main program for closed frequent item set mining

---

1. For each node **n** in a prefix tree with the depth-first traveling:
2. If the length of TID-list for node **n** is less than a support threshold,
3. then remove this node and it's offspring nodes.
4. If the TID-list of this node **n** would be found in the TID-list library,
5. then remove this node and it's offspring nodes.
6. Check the length of TID-list for all children nodes of this node **n**:
7. If there are not any children node's TID list has the same length with parent node **n**,
8. then report the Item set of node **n**, the TID list of this node is recorded in the TID-list library as well.

Algorithm 1 illustrates the framework, including the necessary main step.

Lines 2-3, which are a support-constraint that the frequent item set, must appear in the dataset more frequently than a threshold.

Lines 4-5, according to **Proposition 5**, if the superset of the frequent item set for node **n** has been found before, then it is unnecessary to reach node **n** and the offspring of node **n**.

Lines 6-8, according to **Proposition 4**, if the length of the TID list for all children nodes of node **n** is less than this node **n**, then this node is the maximal one for a TlD list. According to the definition of the closed frequent item set, this node must be reported and the TID list of node **n** recorded in the TID-list library as well, as shown in **Figure S4**.

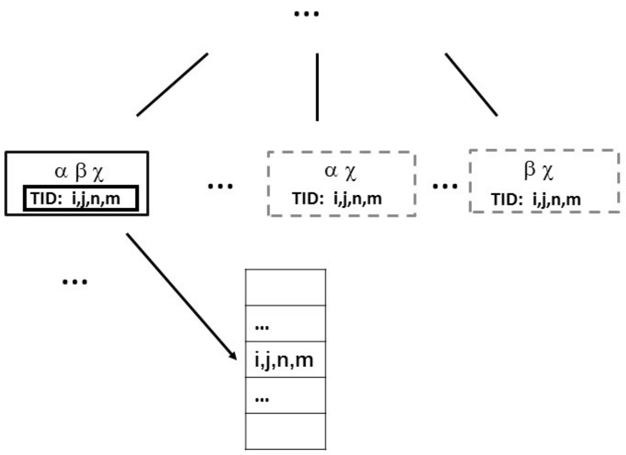

Figure S4 The rule to record the TID-list

If a frequent set has been reported then the TID-list will be recorded in TID-list library. Any node with a TID list which have been recorded are deleted.

The depth of this prefix tree can be very large in a practical application. Depth-first traveling for a prefix tree is implemented by stack architecture instead of recursive call, therefore, large depth for traveling is available.
