# Supplementary figures and images for: CFSP: a collaborative frequent sequence pattern discovery algorithm for nucleic acid sequence classification
Source: PeerJ. 2020 Apr 20;8:e8965. doi: 10.7717/peerj.8965 (PMC7179567; doi:10.7717/peerj.8965)

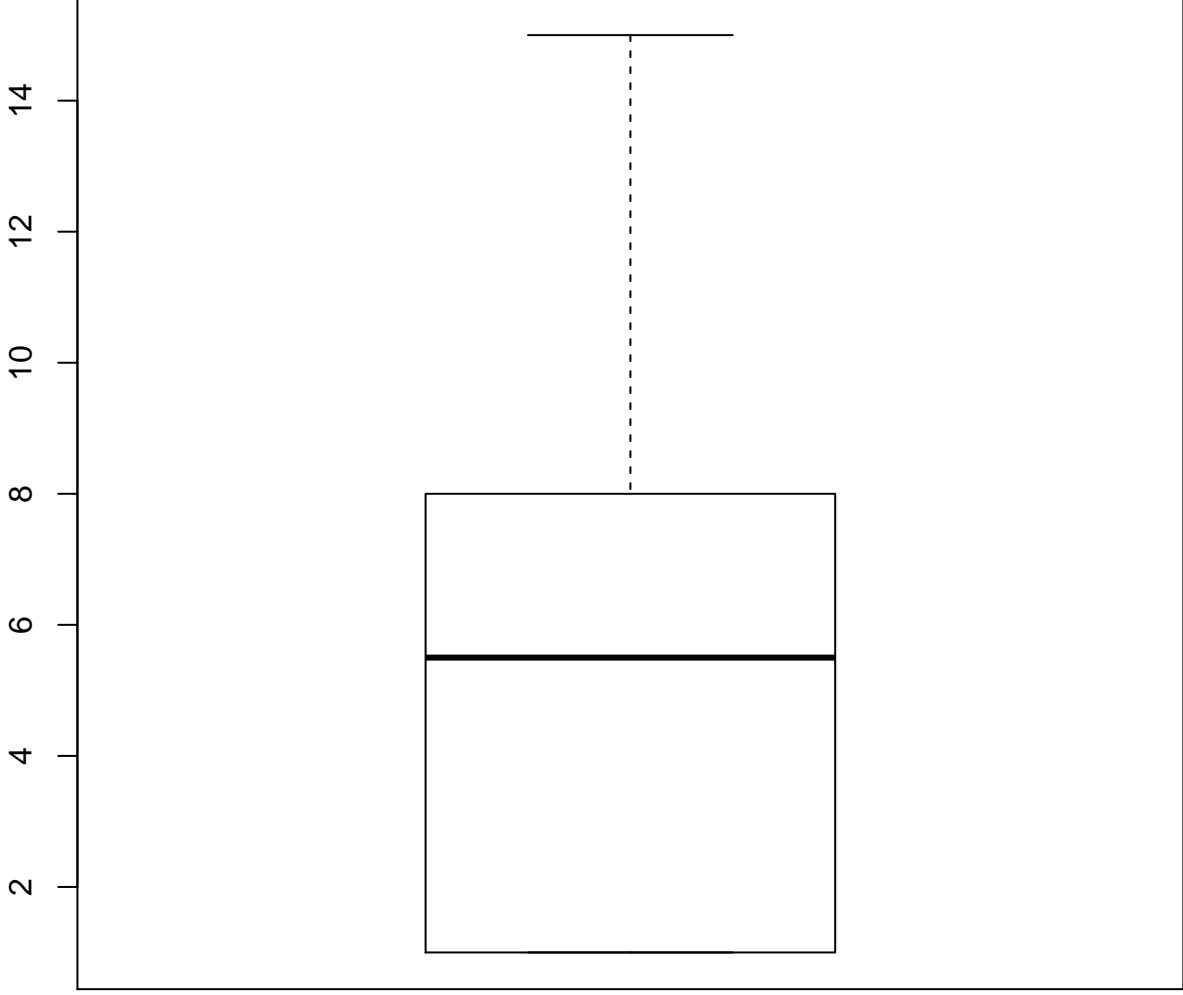

Supplement: Supplemental Information 2 [file peerj-08-8965-s002.zip › ChIP-Seq/SREBF1/Box.pdf]

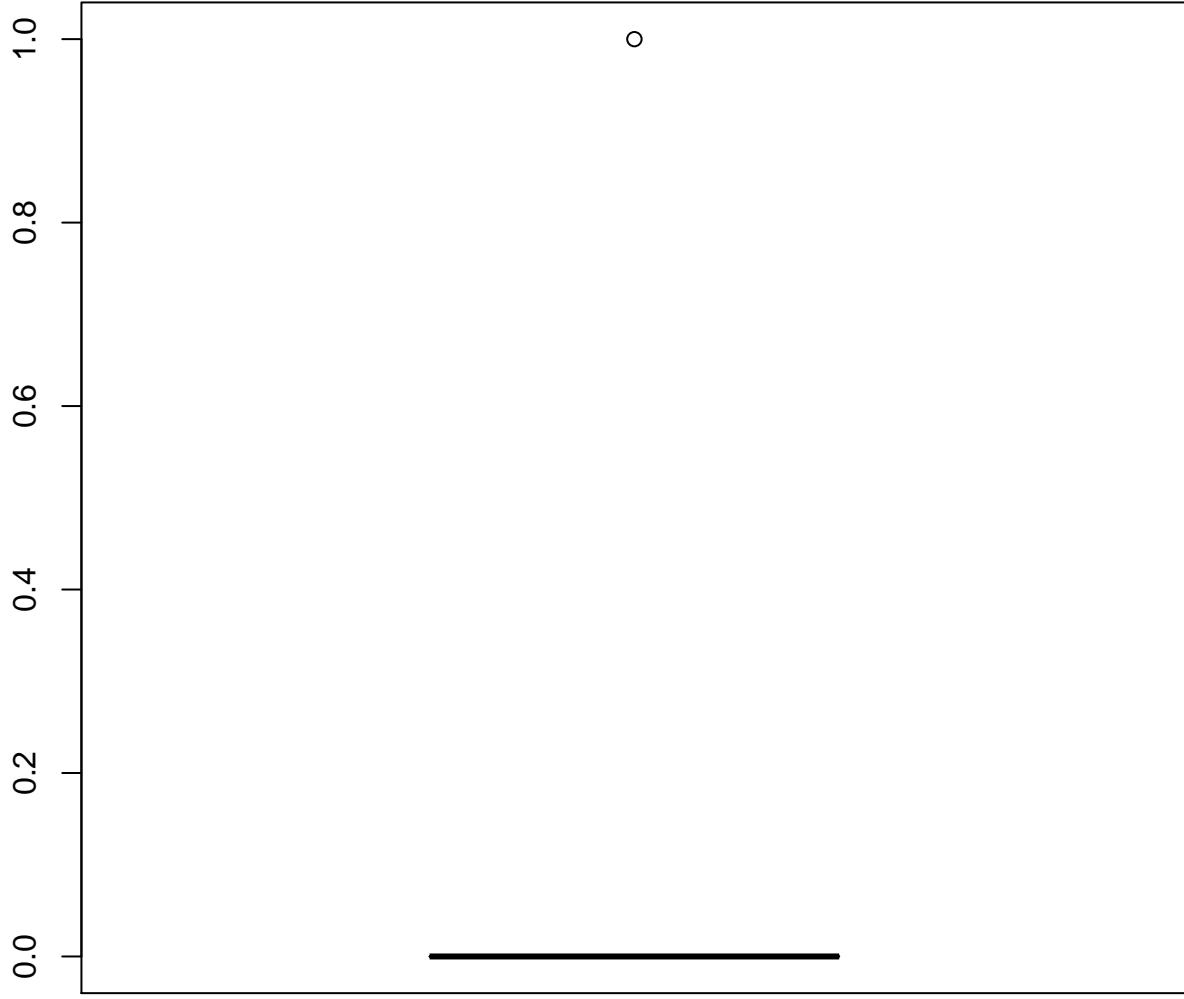

Supplement: Supplemental Information 2 [file peerj-08-8965-s002.zip › ChIP-Seq/SREBF1/Box_.pdf]

QQ-plot for all 1,065,392 p-values

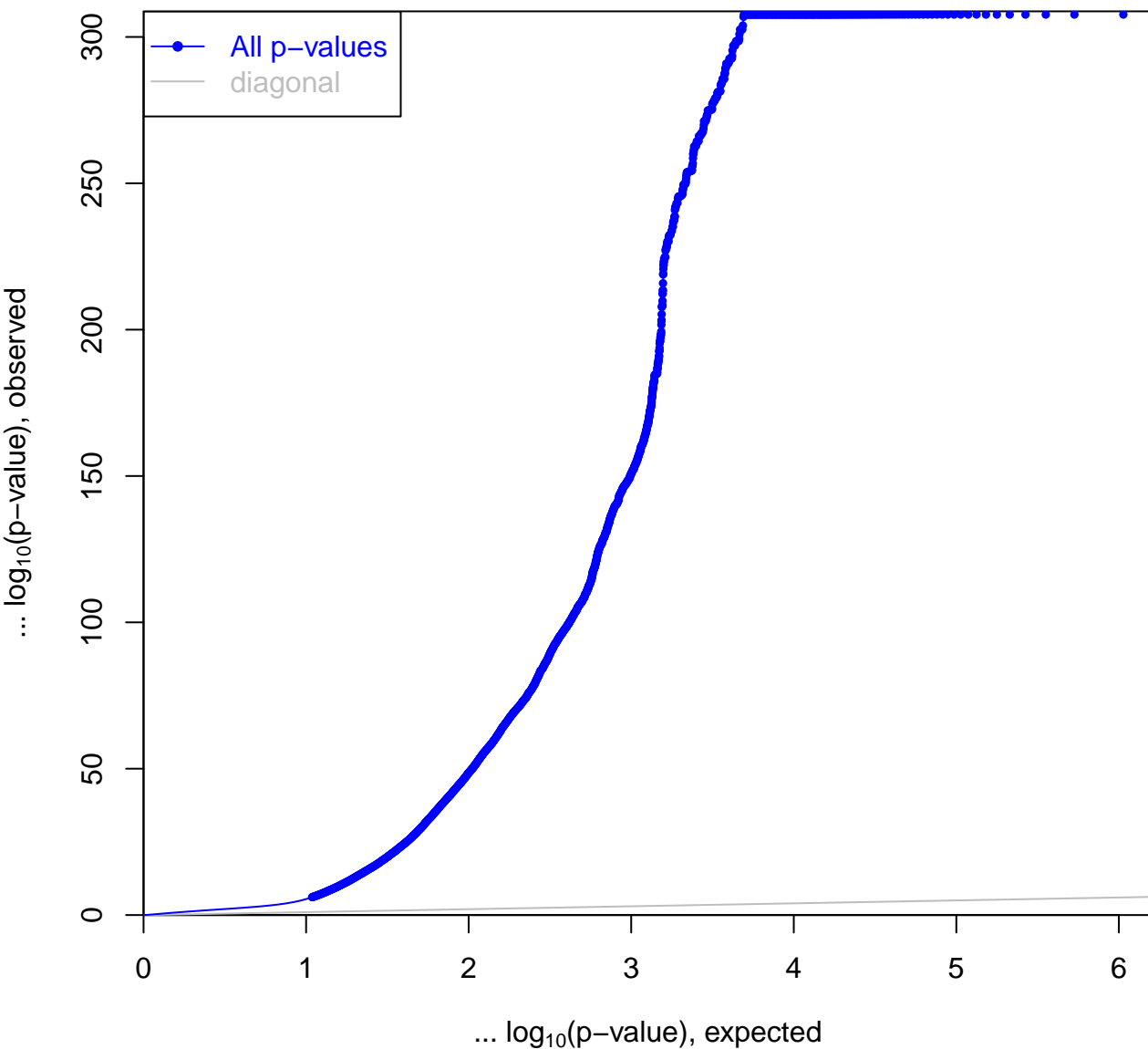

Supplement: Supplemental Information 2 [file peerj-08-8965-s002.zip › ChIP-Seq/SREBF1/Co_eqtl.pdf]

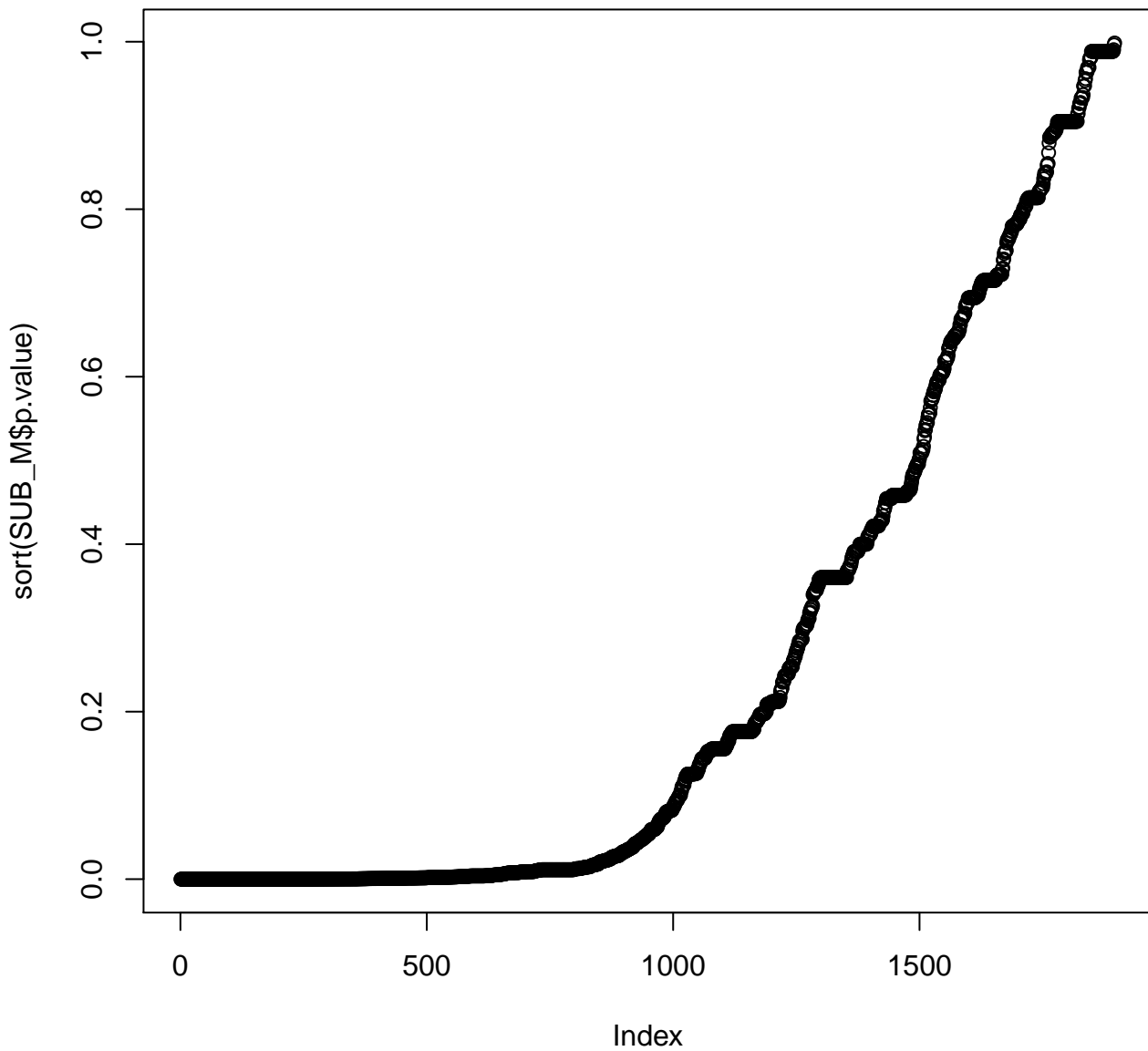

Supplement: Supplemental Information 2 [file peerj-08-8965-s002.zip › ChIP-Seq/SREBF1/P.value (2).pdf]

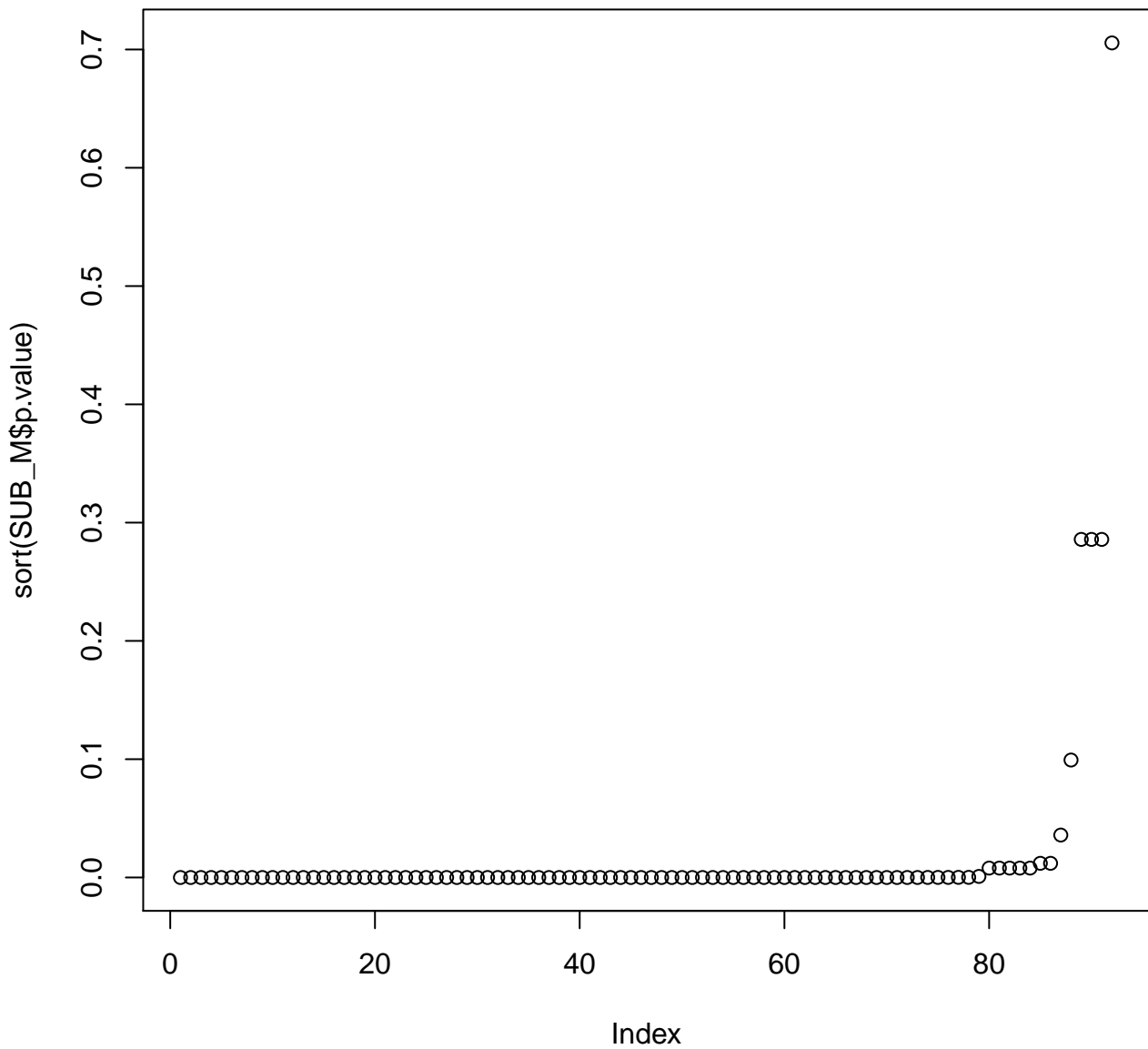

Supplement: Supplemental Information 2 [file peerj-08-8965-s002.zip › ChIP-Seq/SREBF1/P.value (3).pdf]

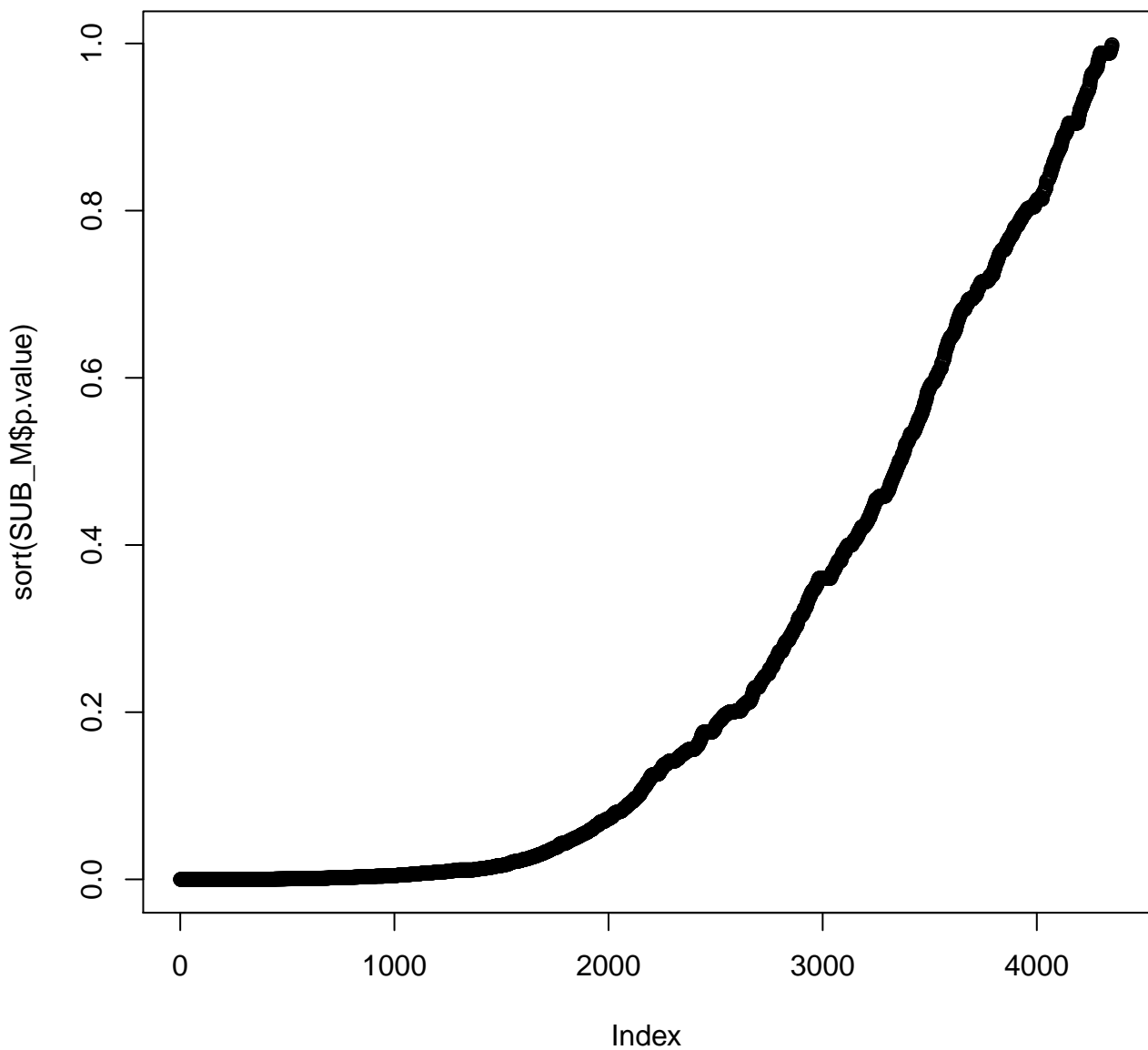

Supplement: Supplemental Information 2 [file peerj-08-8965-s002.zip › ChIP-Seq/SREBF1/P.value.pdf]

QQ-plot for all 998,805 p-values

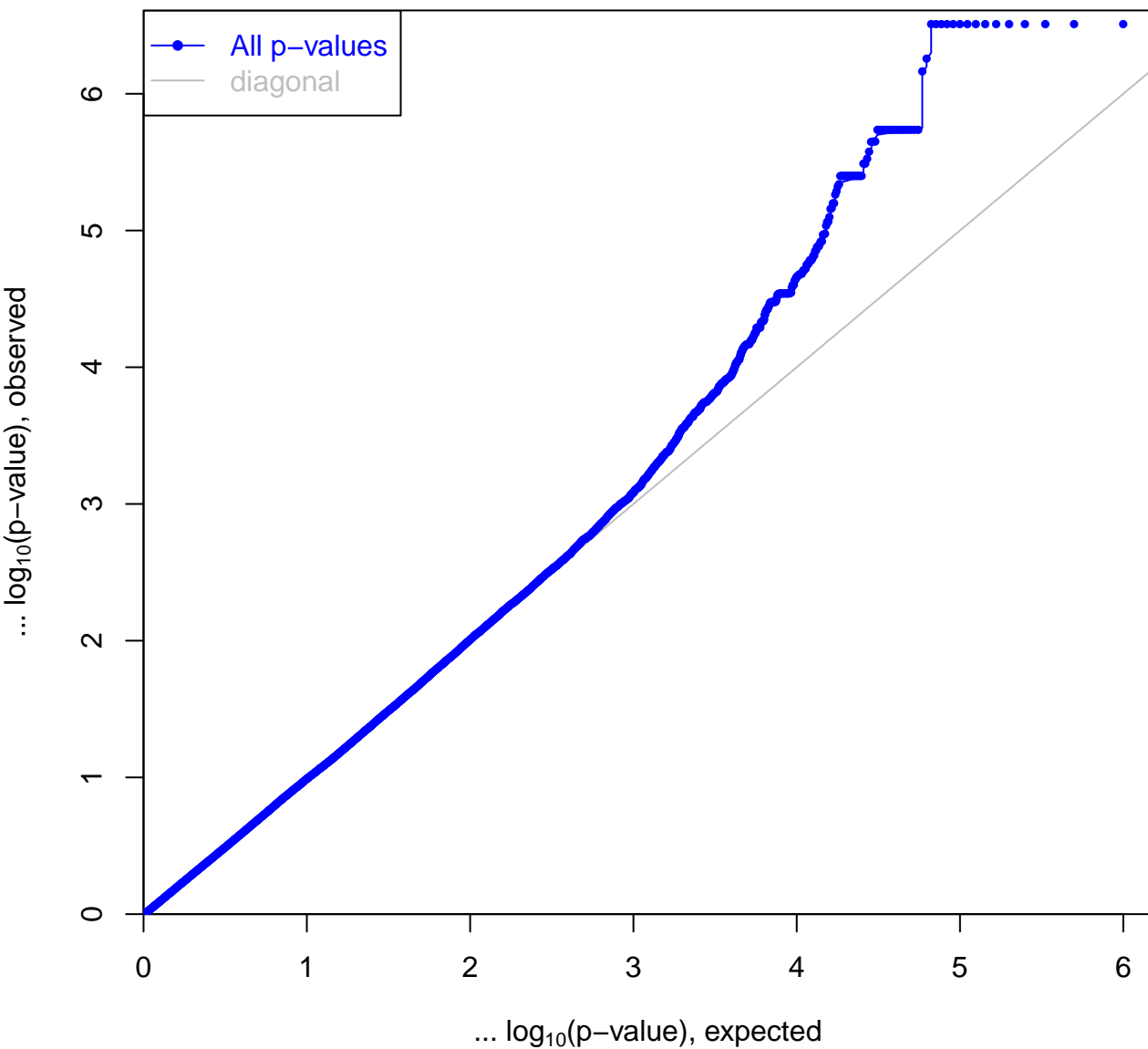

Supplement: Supplemental Information 2 [file peerj-08-8965-s002.zip › ChIP-Seq/SREBF1/co.eQTL (2).pdf]

Histogram of X

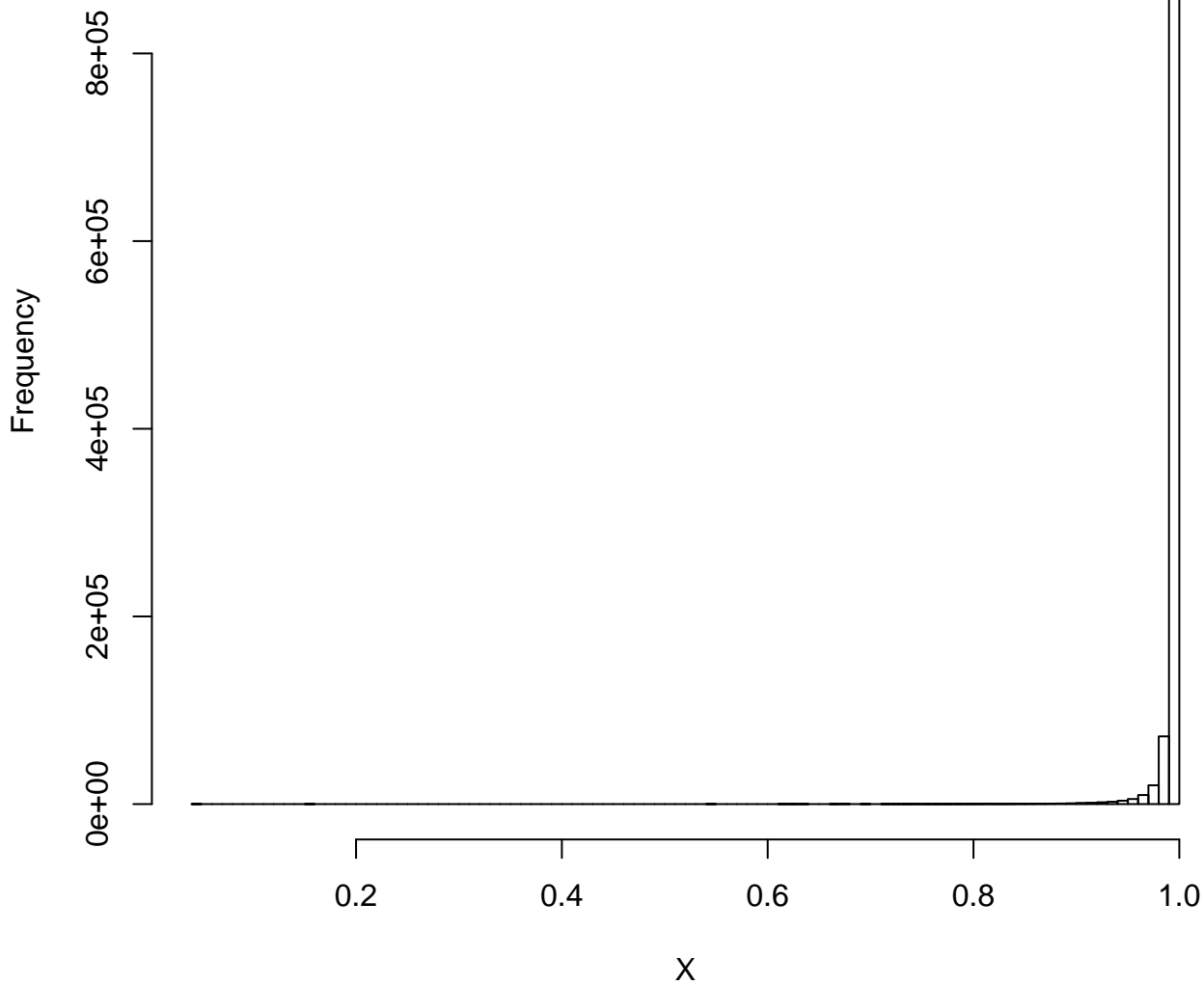

Supplement: Supplemental Information 2 [file peerj-08-8965-s002.zip › ChIP-Seq/SREBF1/hist (2).pdf]

Histogram of  $M_{[, 2]}$

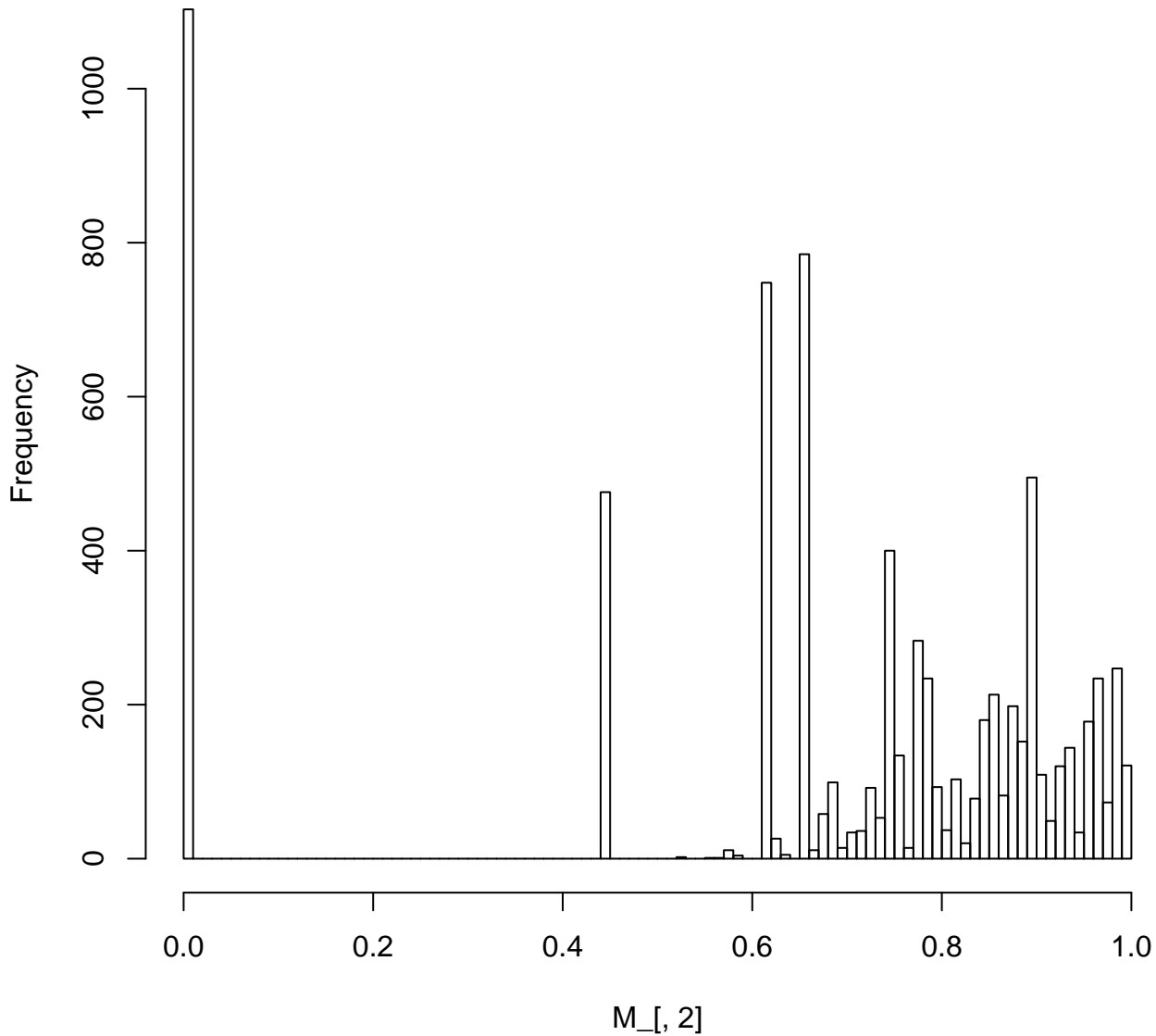

Supplement: Supplemental Information 2 [file peerj-08-8965-s002.zip › ChIP-Seq/SREBF1/hist (3).pdf]

Histogram of X

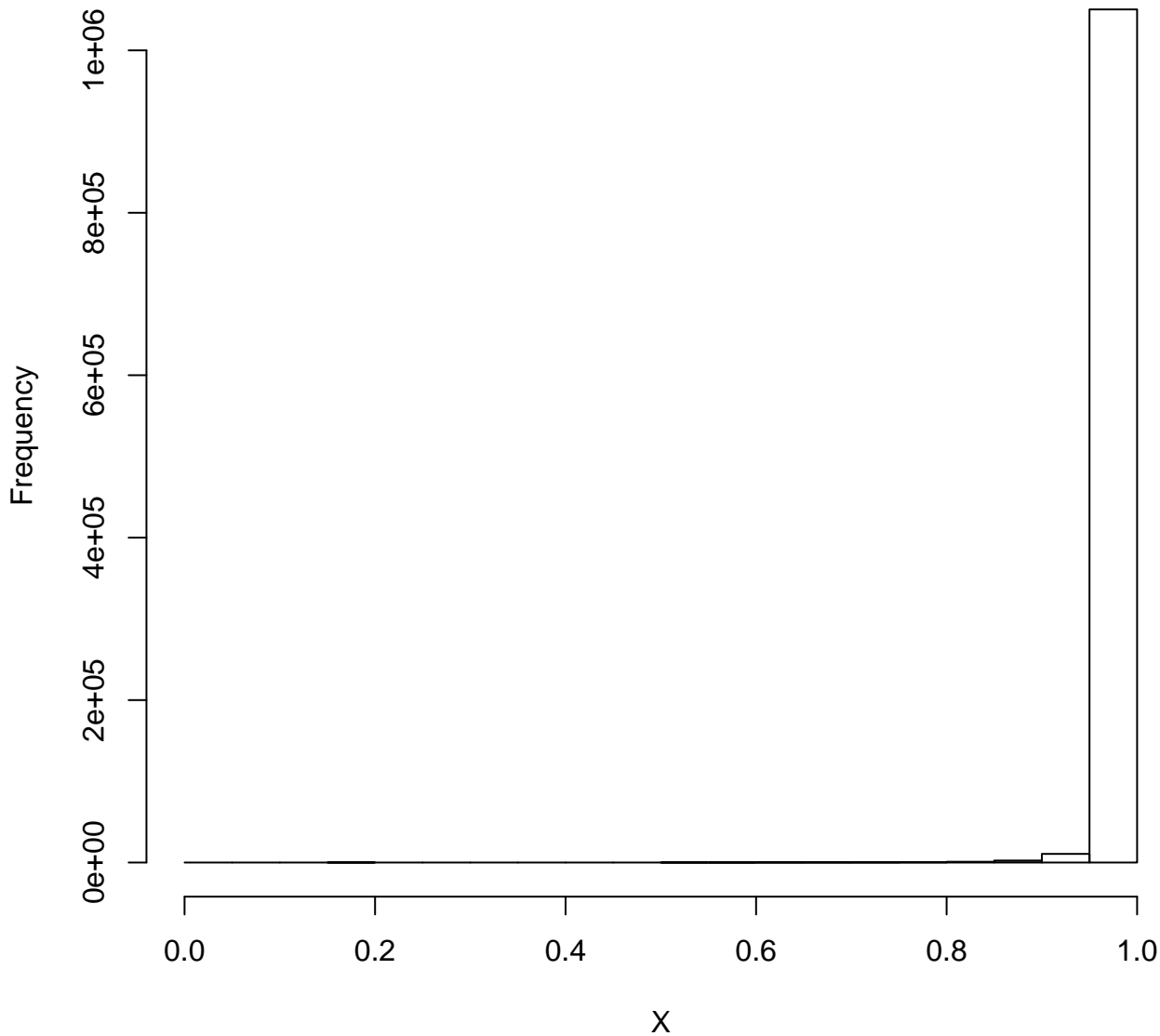

Supplement: Supplemental Information 2 [file peerj-08-8965-s002.zip › ChIP-Seq/SREBF1/hist.pdf]

QQ-plot for all 1,065,392 p-values

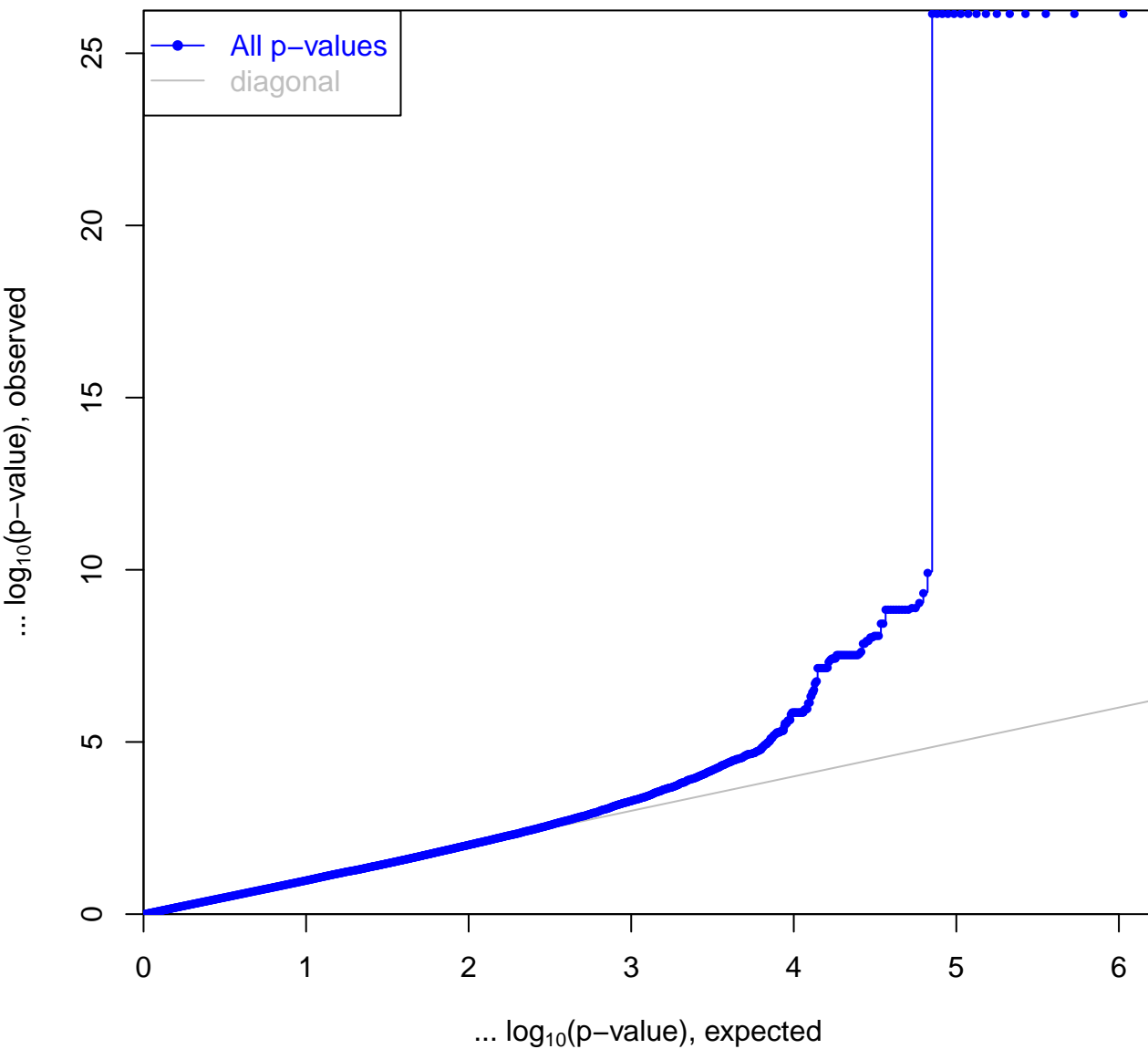

Supplement: Supplemental Information 2 [file peerj-08-8965-s002.zip › ChIP-Seq/SREBF1/meq_.pdf]

QQ-plot for all 998,805 p-values

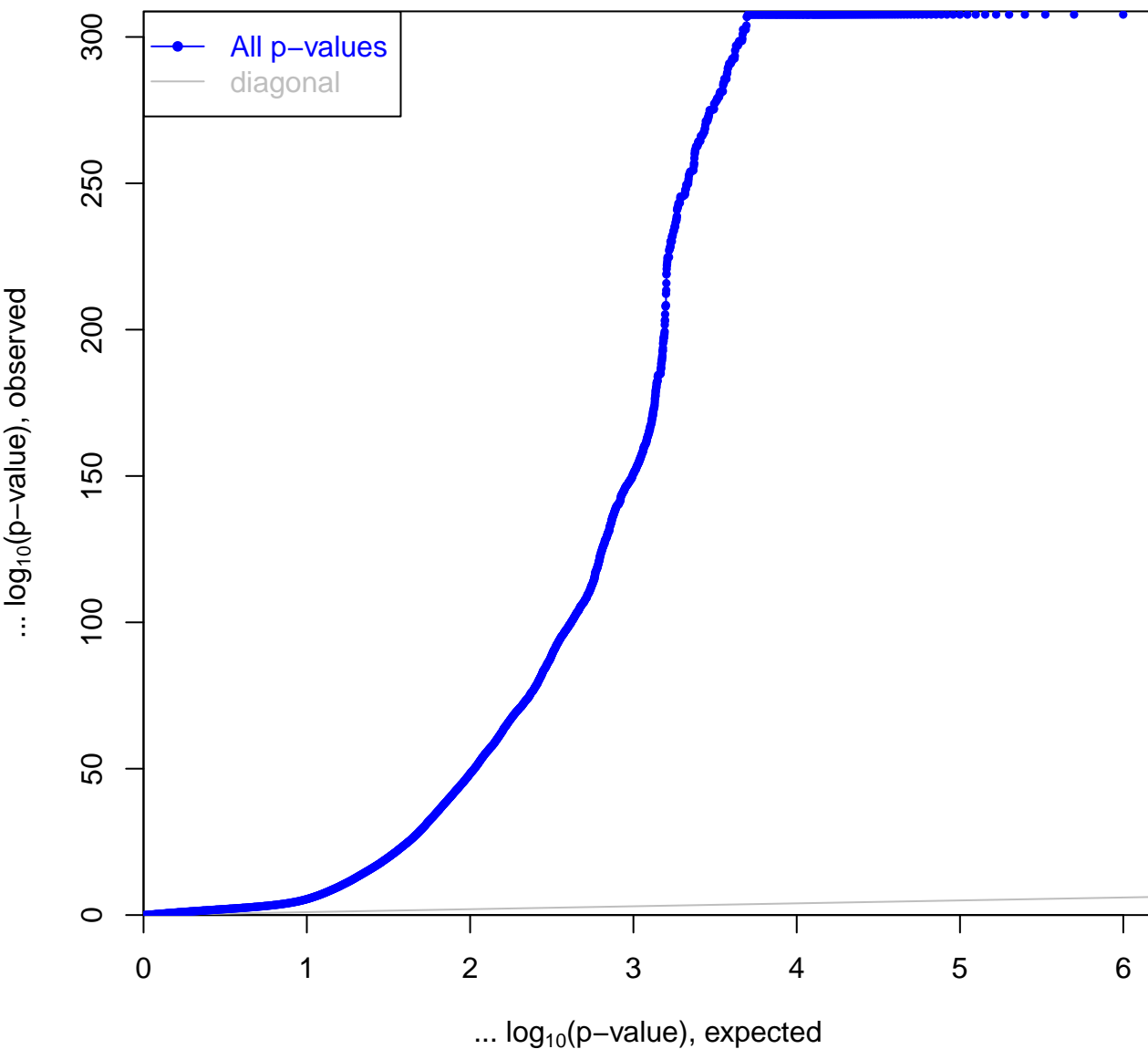

Supplement: Supplemental Information 2 [file peerj-08-8965-s002.zip › ChIP-Seq/SREBF1/paise_EQTL.pdf]

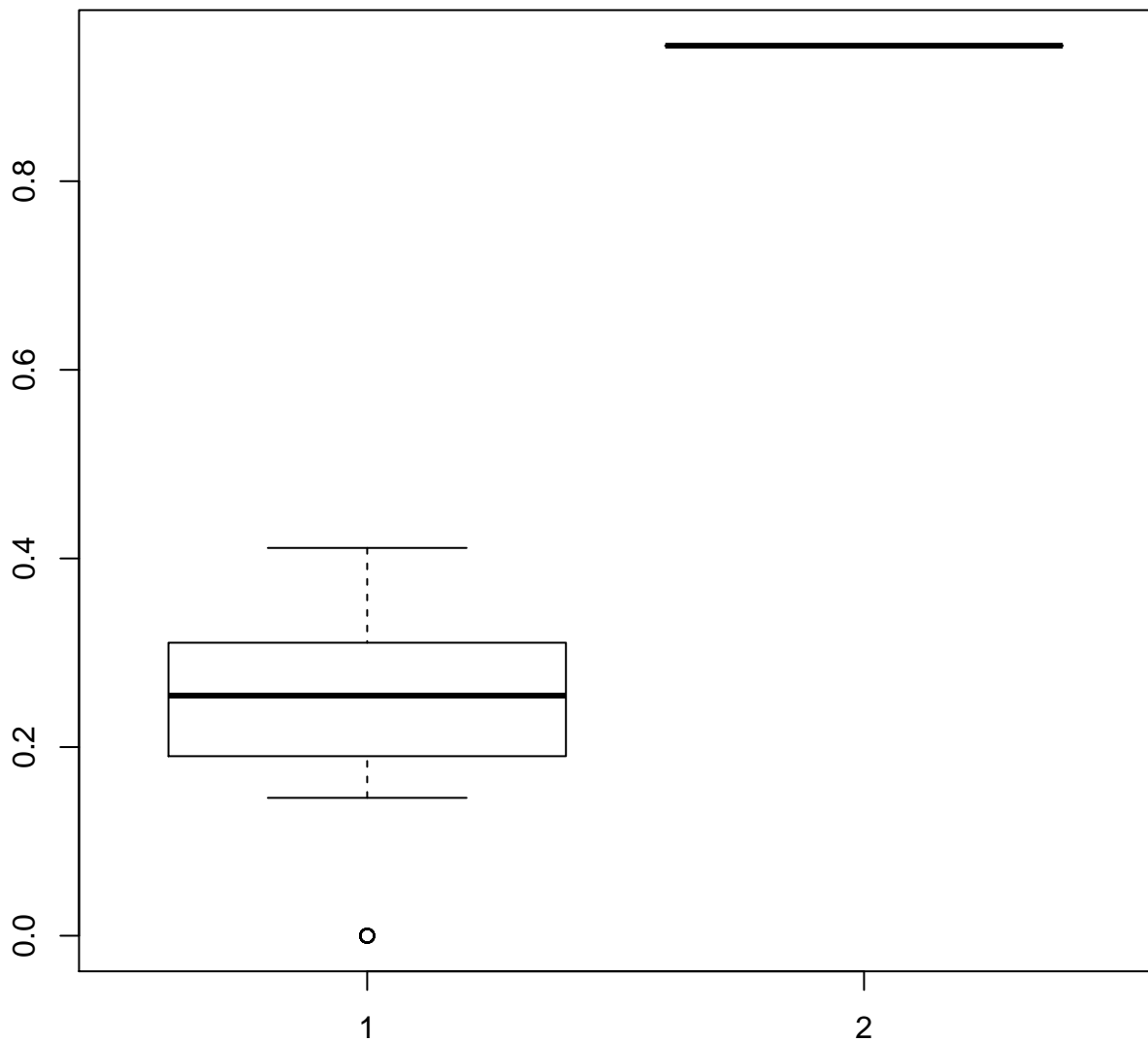

Supplement: Supplemental Information 2 [file peerj-08-8965-s002.zip › ChIP-Seq/SREBF1/x_Y.pdf]

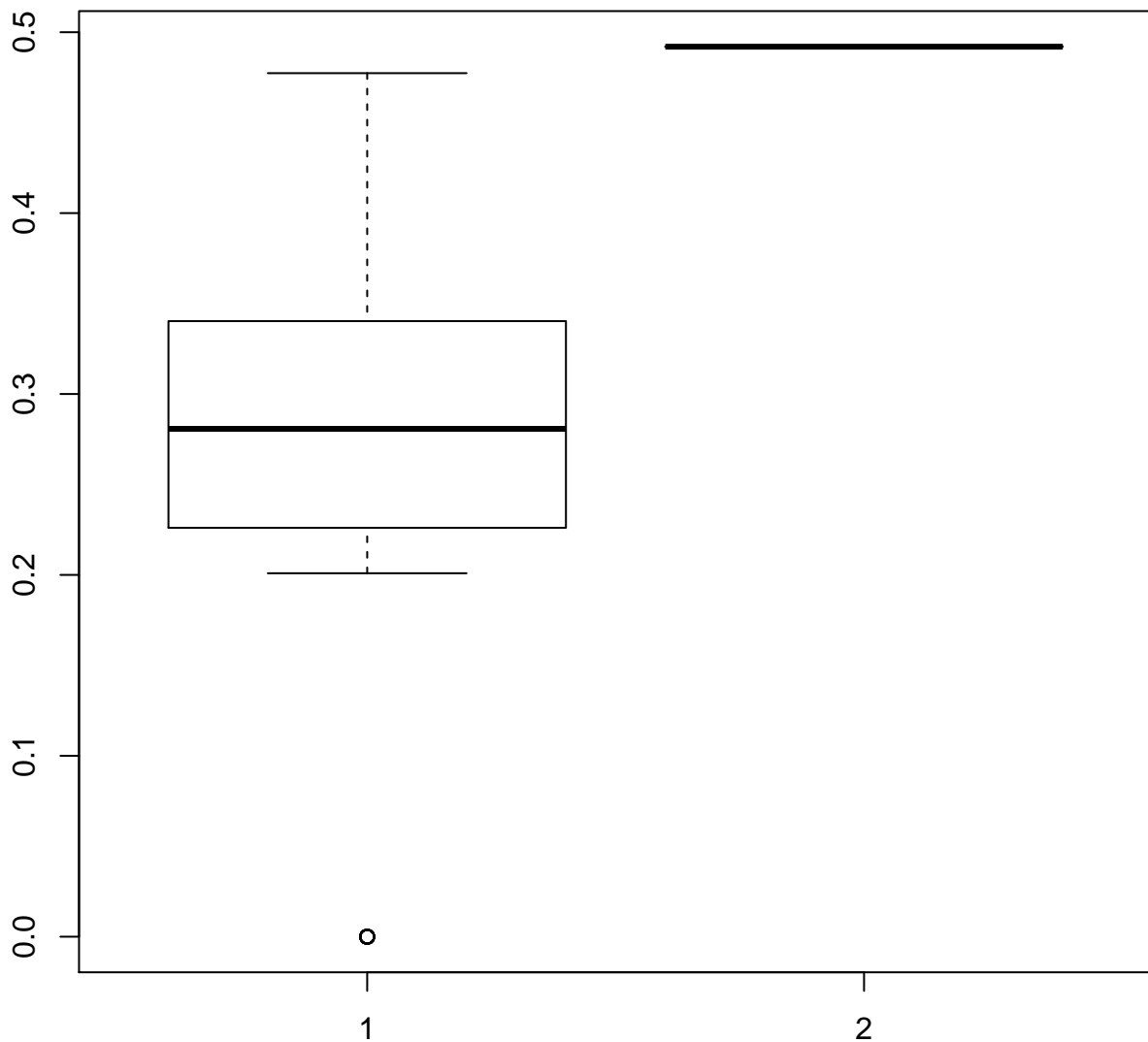

Supplement: Supplemental Information 2 [file peerj-08-8965-s002.zip › ChIP-Seq/SREBF1/x_Y_1.pdf]

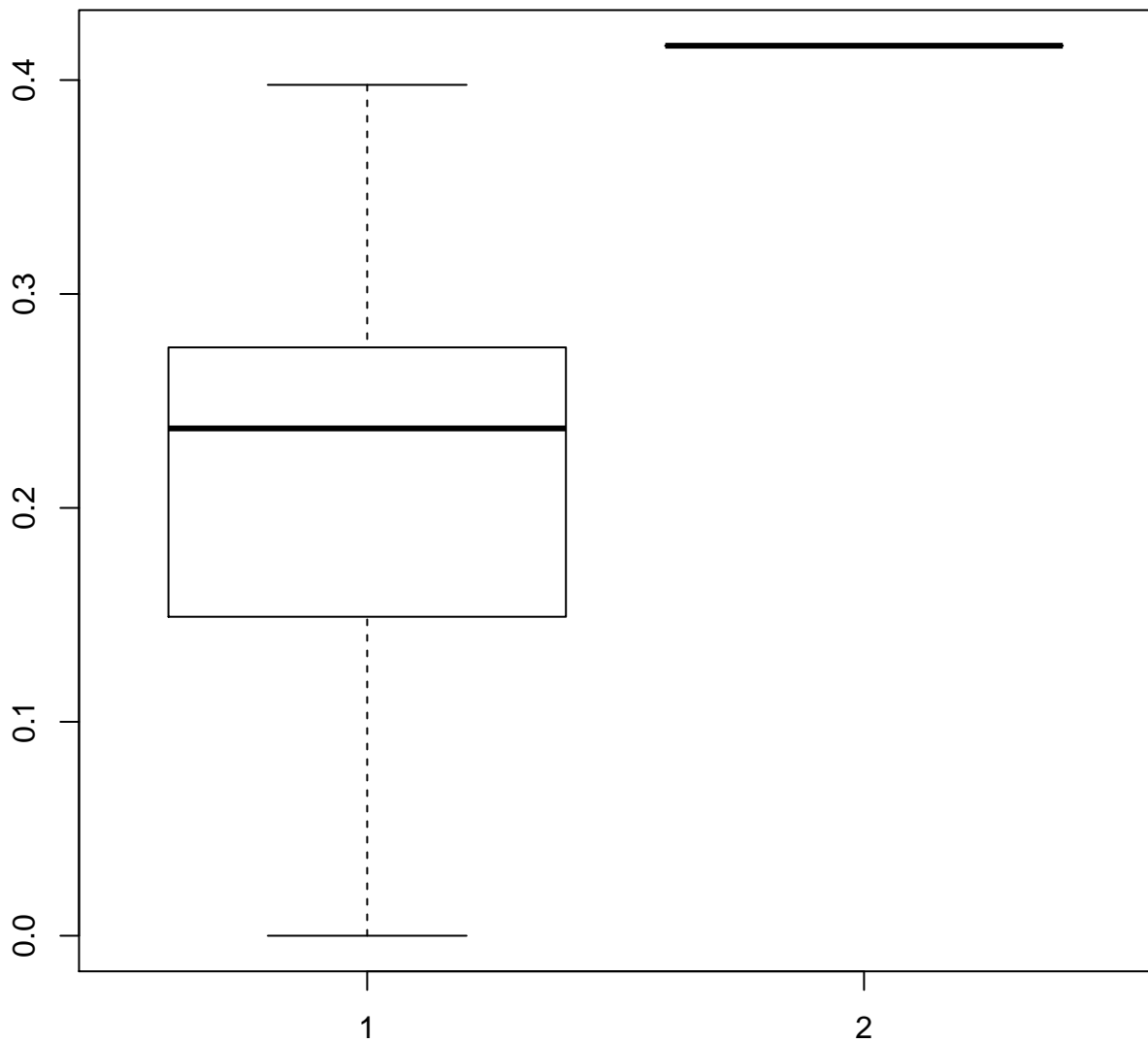

Supplement: Supplemental Information 2 [file peerj-08-8965-s002.zip › ChIP-Seq/SREBF1/x_Y_10.pdf]

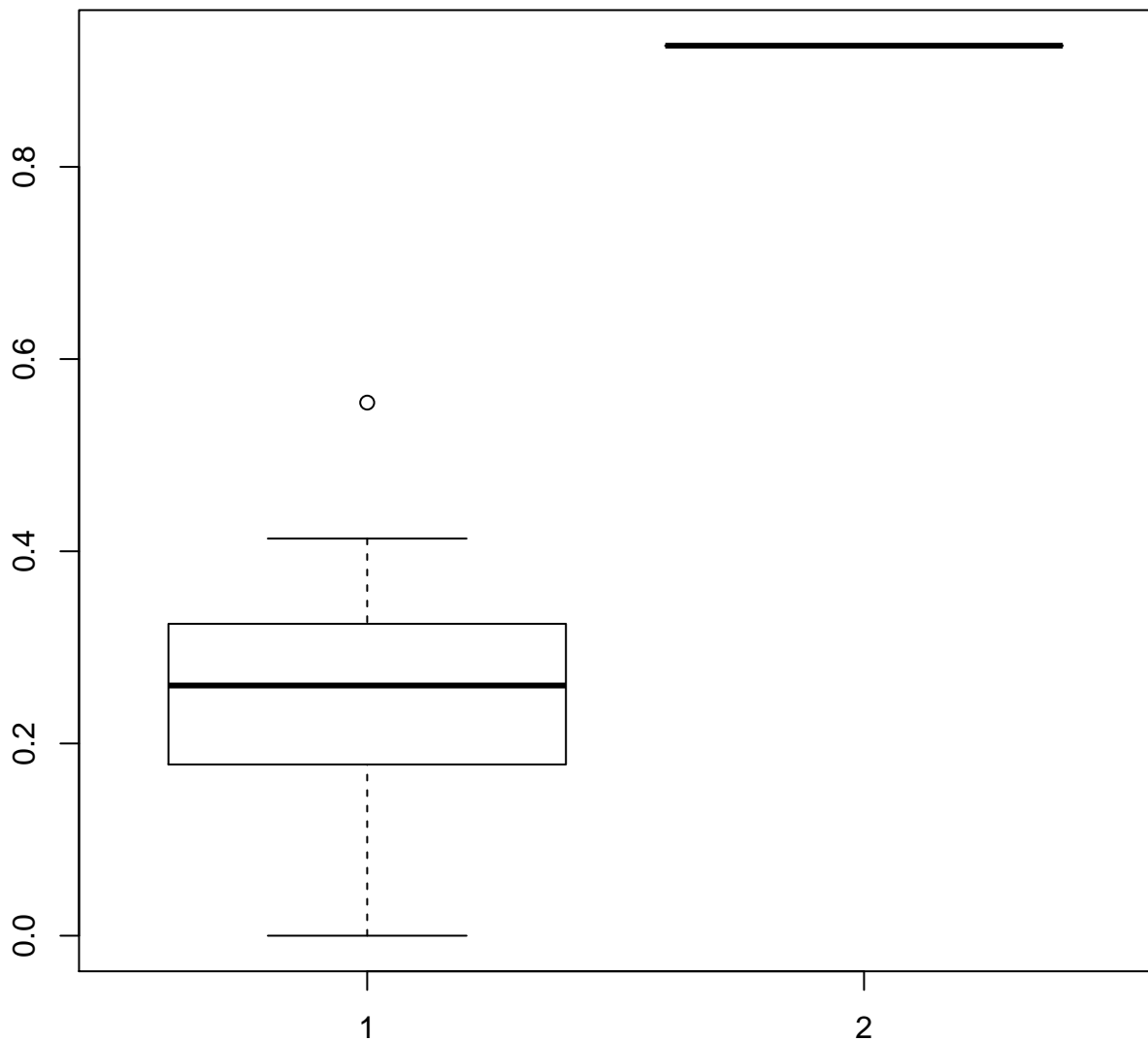

Supplement: Supplemental Information 2 [file peerj-08-8965-s002.zip › ChIP-Seq/SREBF1/x_Y_11.pdf]

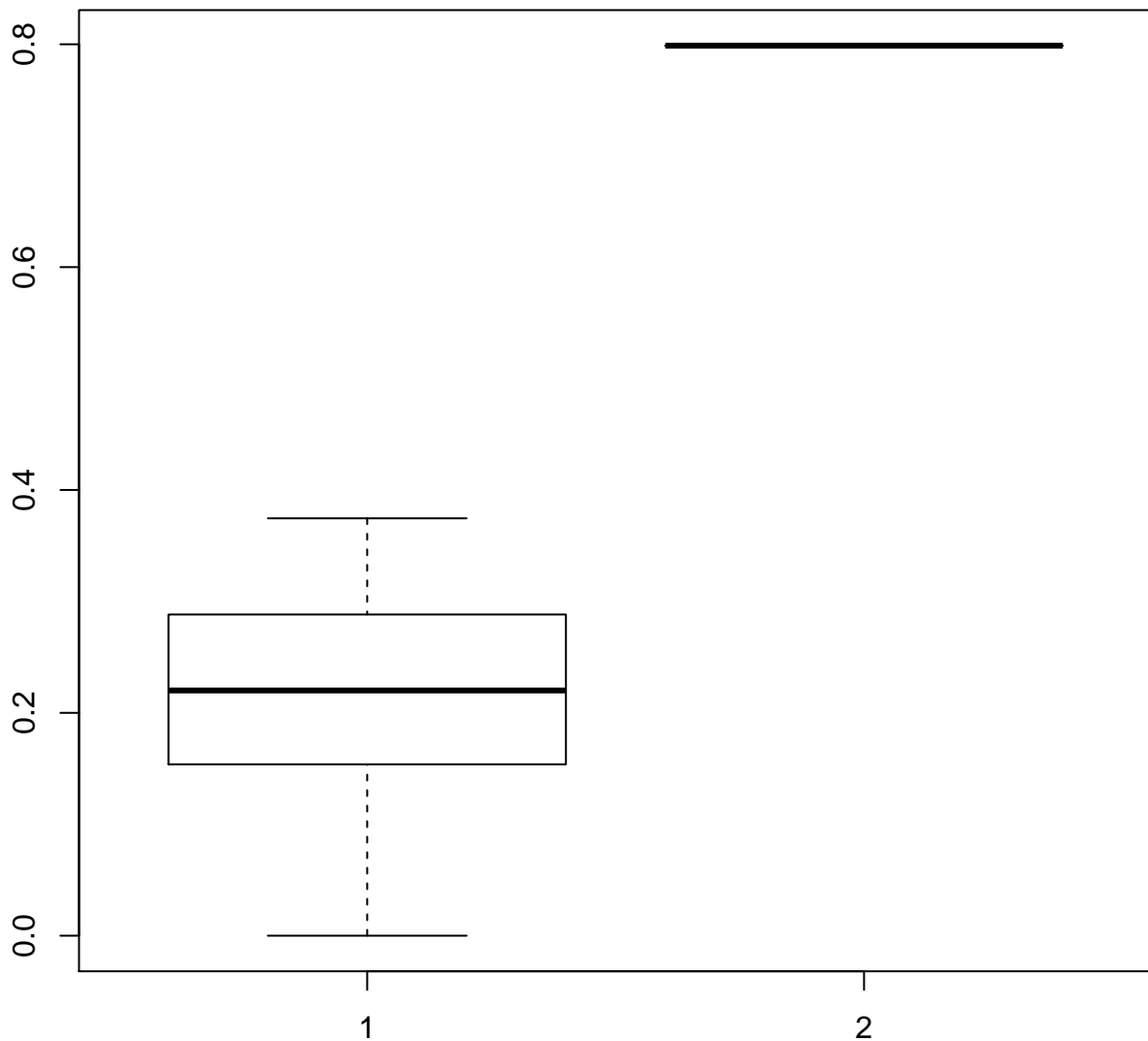

Supplement: Supplemental Information 2 [file peerj-08-8965-s002.zip › ChIP-Seq/SREBF1/x_Y_12.pdf]

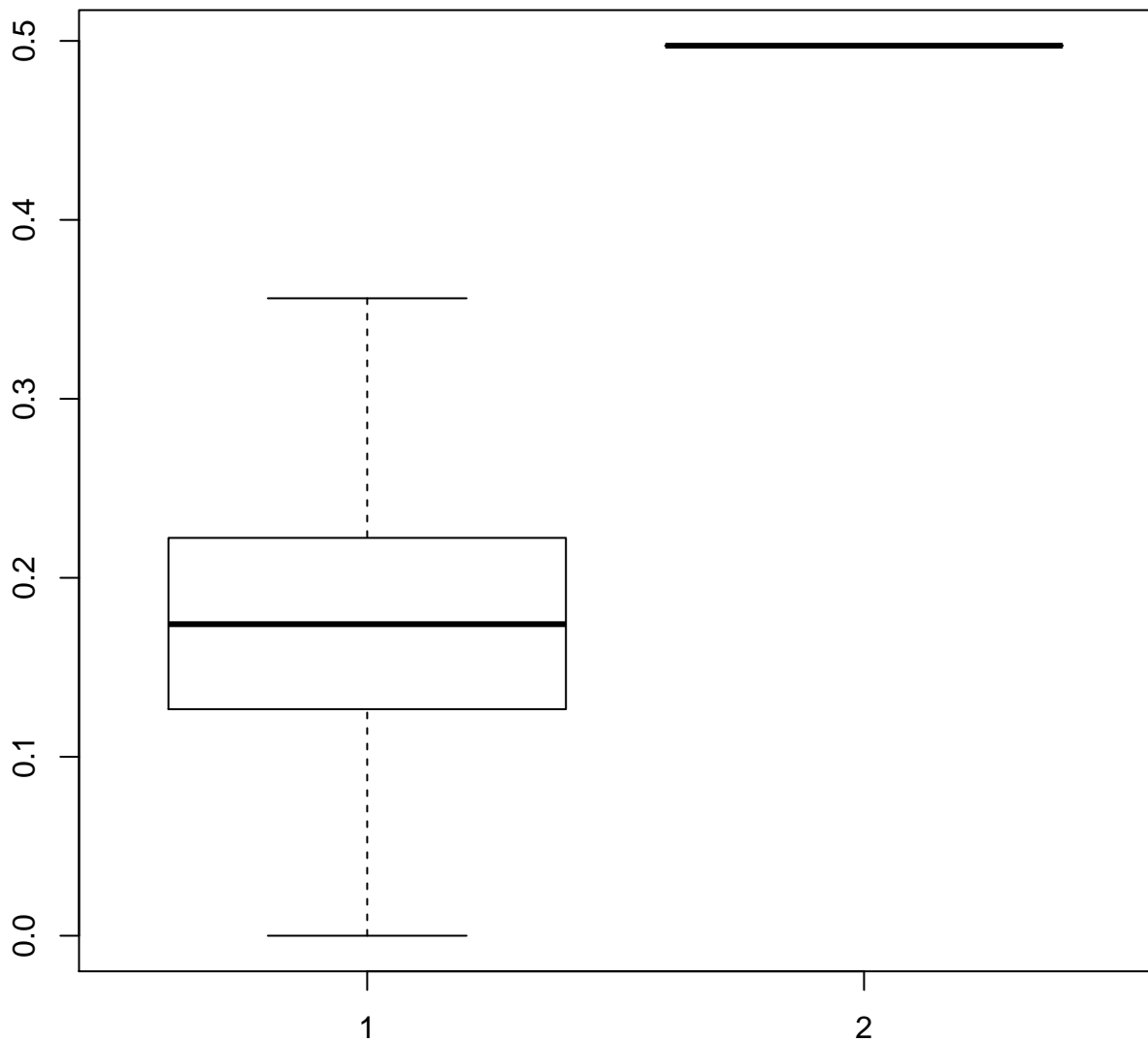

Supplement: Supplemental Information 2 [file peerj-08-8965-s002.zip › ChIP-Seq/SREBF1/x_Y_14.pdf]

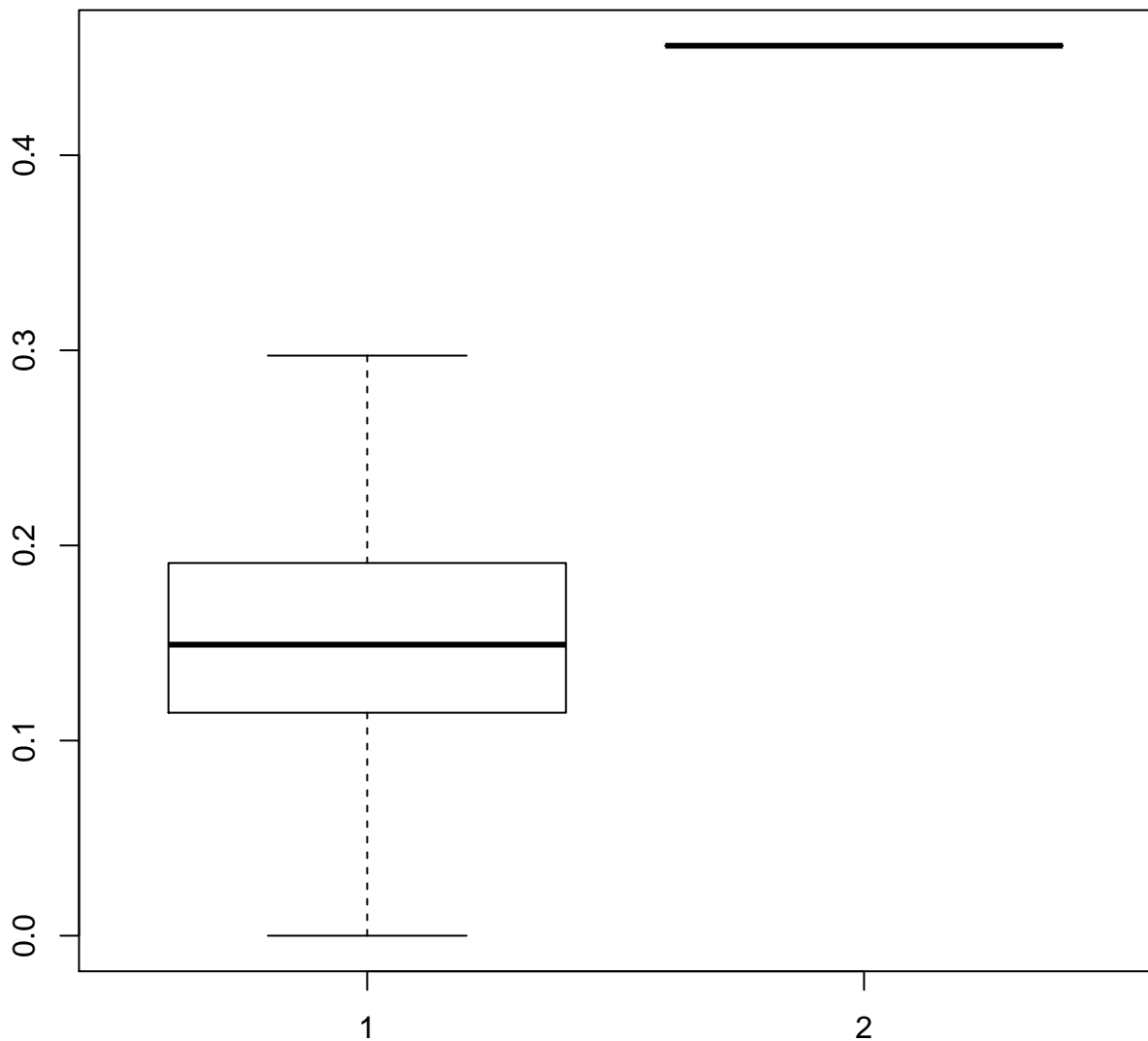

Supplement: Supplemental Information 2 [file peerj-08-8965-s002.zip › ChIP-Seq/SREBF1/x_Y_5.pdf]

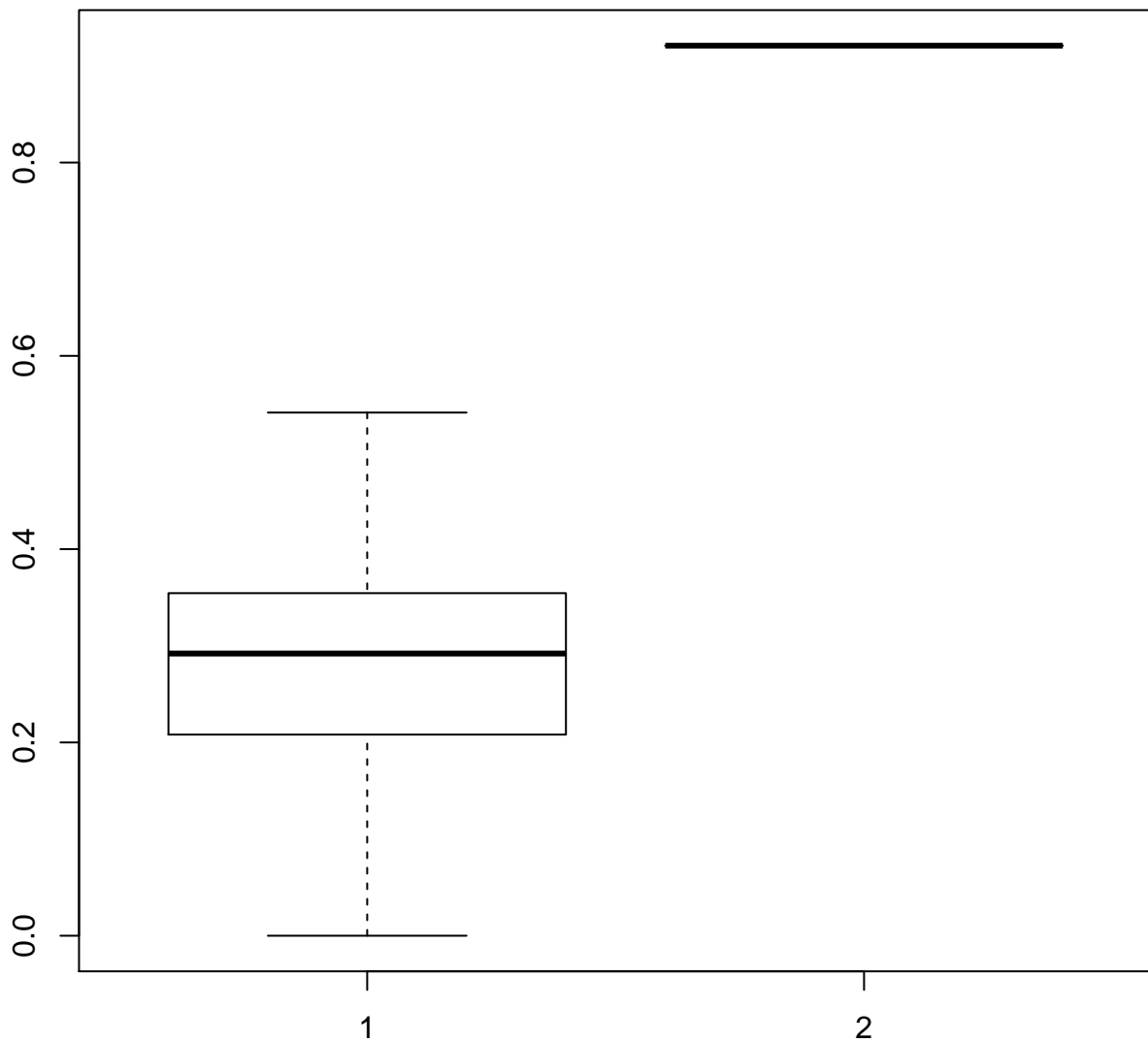

Supplement: Supplemental Information 2 [file peerj-08-8965-s002.zip › ChIP-Seq/SREBF1/x_Y_6.pdf]

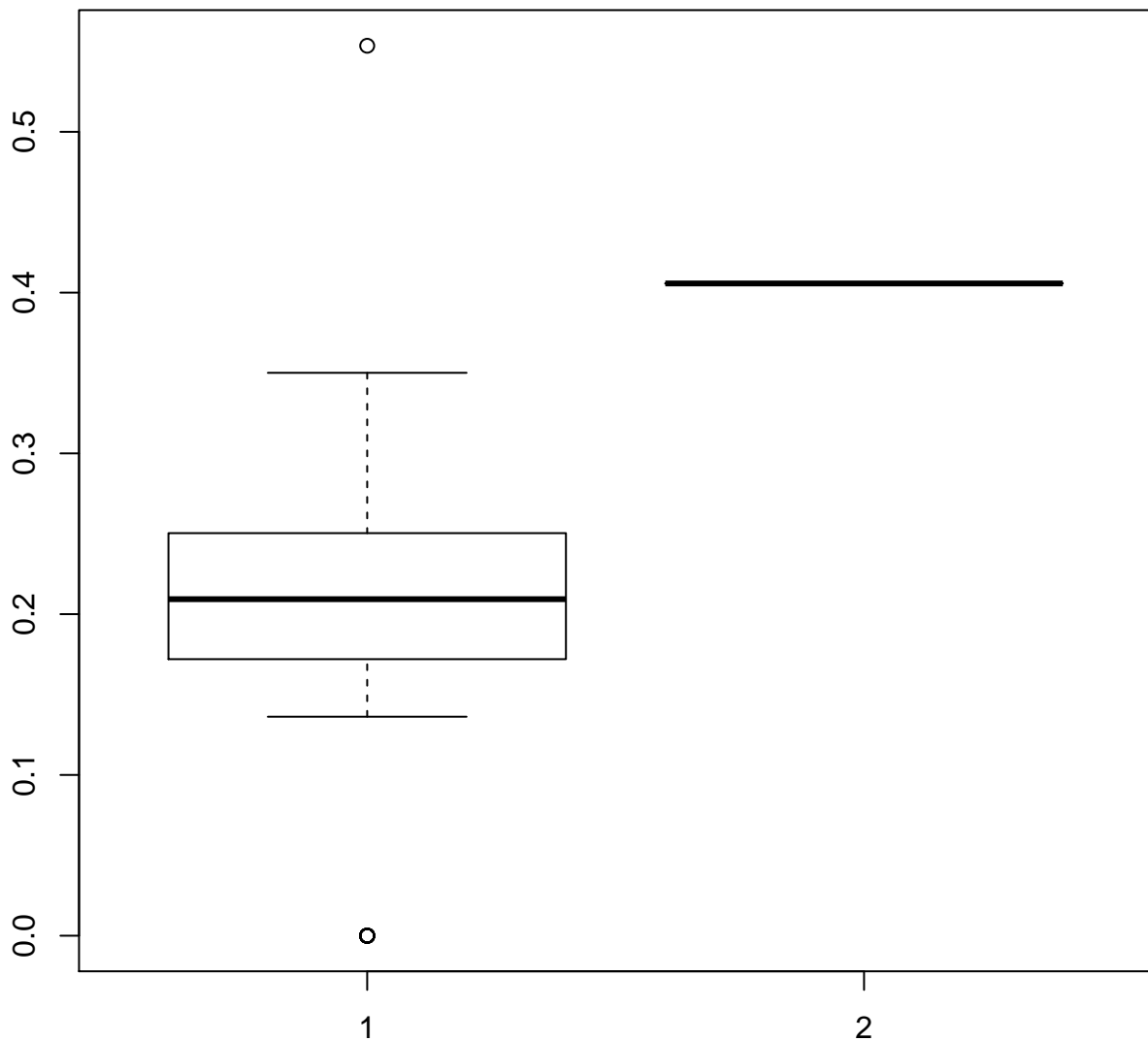

Supplement: Supplemental Information 2 [file peerj-08-8965-s002.zip › ChIP-Seq/SREBF1/x_Y_7.pdf]

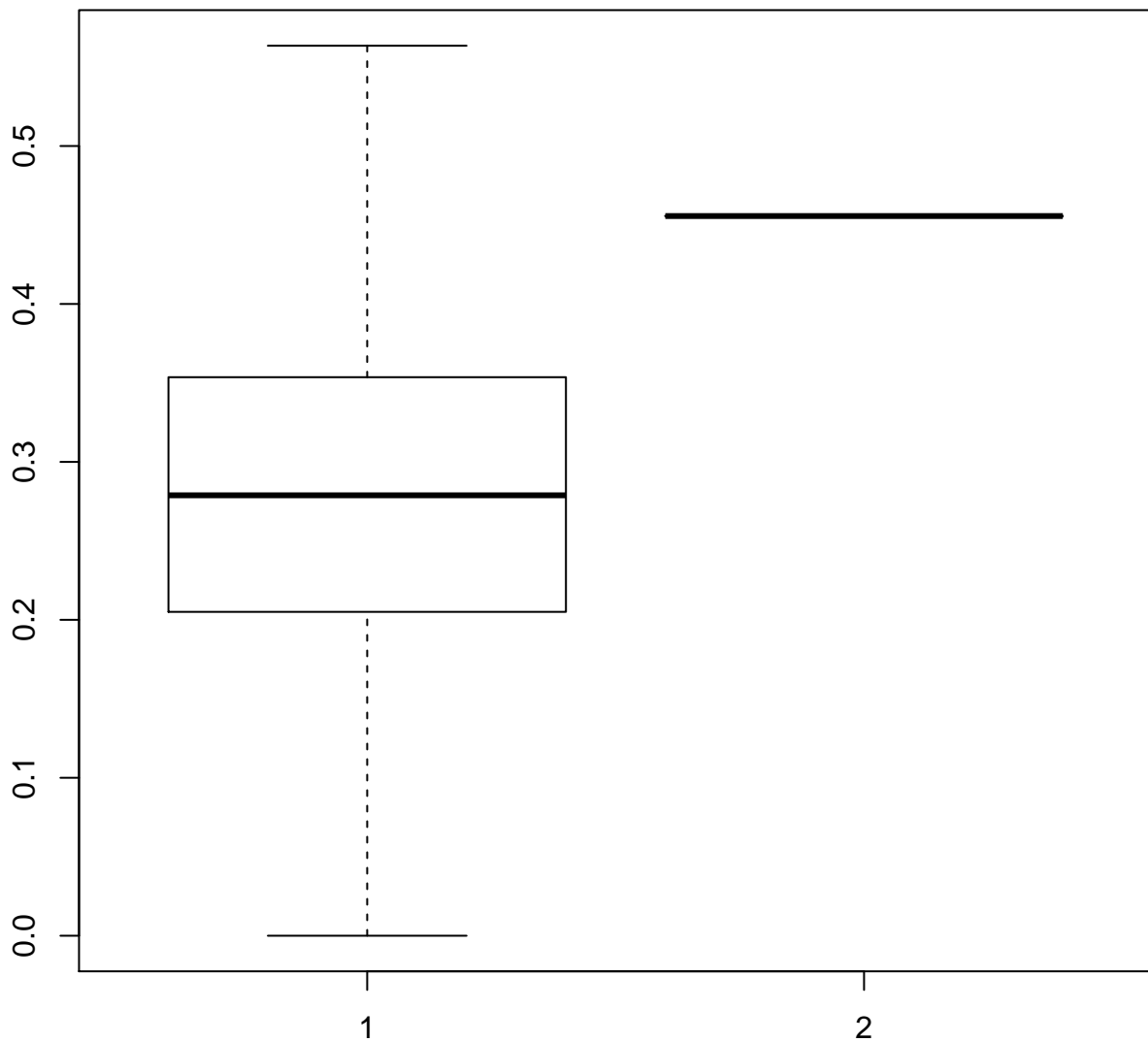

Supplement: Supplemental Information 2 [file peerj-08-8965-s002.zip › ChIP-Seq/SREBF1/x_Y_8.pdf]

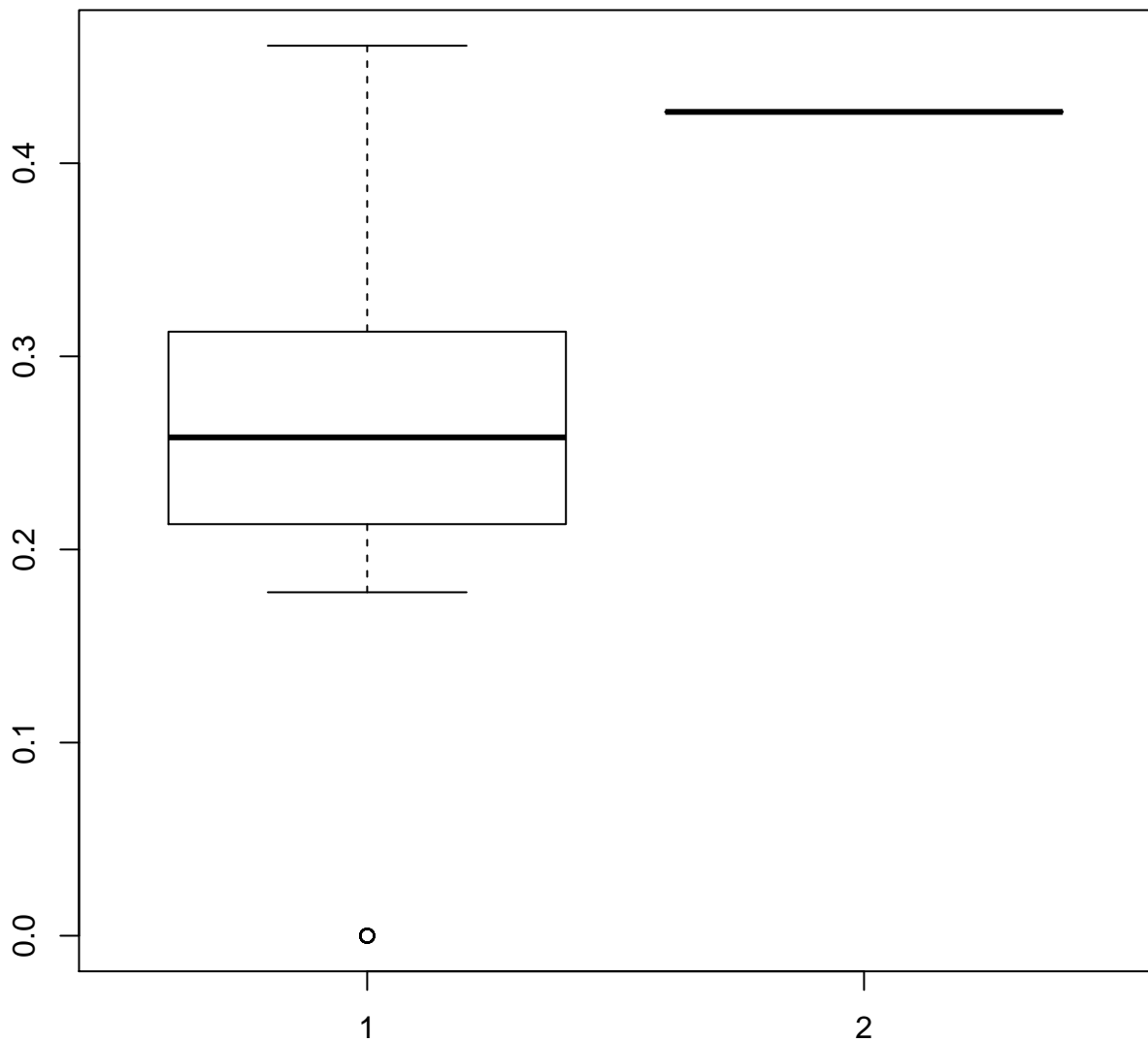

Supplement: Supplemental Information 2 [file peerj-08-8965-s002.zip › ChIP-Seq/SREBF1/x_Y_9.pdf]
